# Nanotechnology-Abetted Astaxanthin Formulations in Multimodel Therapeutic and Biomedical Applications

**DOI:** 10.1021/acs.jmedchem.1c01144

**Published:** 2021-12-17

**Authors:** Zohreh Jafari, Ashkan Bigham, Sahar Sadeghi, Sayed Mehdi Dehdashti, Navid Rabiee, Alireza Abedivash, Mojtaba Bagherzadeh, Behzad Nasseri, Hassan Karimi-Maleh, Esmaeel Sharifi, Rajender S. Varma, Pooyan Makvandi

**Affiliations:** † Department of Medical Biotechnology, School of Advanced Technologies in Medicine, Shahid Beheshti University of Medical Sciences, 19857-17443 Tehran, Iran; ‡ Institute of Polymers, Composites and Biomaterials - National Research Council (IPCB-CNR), Viale J.F. Kennedy 54 - Mostra D’Oltremare pad. 20, 80125 Naples, Italy; § Department of Medical Biotechnology, School of Advanced Technologies in Medicine, Shahid Beheshti University of Medical Sciences, 19857-17443 Tehran, Iran; Δ Cellular and Molecular Biology Research Center, Shahid Beheshti University of Medical Sciences, 19857-17443 Tehran, Iran; ∑ Department of Chemistry, Sharif University of Technology, 11155-9161 Tehran, Iran; ϒ Department of Physics, Sharif University of Technology, 11155-9161 Tehran, Iran; σ School of Engineering, Macquarie University, Sydney, New South Wales 2109, Australia; ∧ Department of Basic Sciences, Sari Agricultural Sciences and Natural Resources University, 48181-68984 Sari, Iran; & Department of Medical Biotechnology, Faculty of Advance Medical Sciences, Tabriz University of Medical Sciences, 51664 Tabriz, Iran; ″ School of Resources and Environment, University of Electronic Science and Technology of China, P.O. Box 611731, Xiyuan Avenue, 610054 Chengdu, PR China; ∥ Department of Chemical Engineering, Laboratory of Nanotechnology, Quchan University of Technology, 94771-67335 Quchan, Iran; ⊥ Department of Chemical Sciences, University of Johannesburg, P.O. Box 17011, Doornfontein Campus, 2028, 2006 Johannesburg, South Africa; ⧧ Department of Tissue Engineering and Biomaterials, School of Advanced Medical Sciences and Technologies, Hamadan University of Medical Sciences, 6517838736 Hamadan, Iran; ± Regional Centre of Advanced Technologies and Materials, Czech Advanced Technology and Research Institute, Palacky University, Šlechtitelů 27, 78371 Olomouc, Czech Republic; # Centre for Materials Interfaces, Istituto Italiano di Tecnologia, viale Rinaldo Piaggio 34, 56025 Pontedera, Pisa, Italy

## Abstract

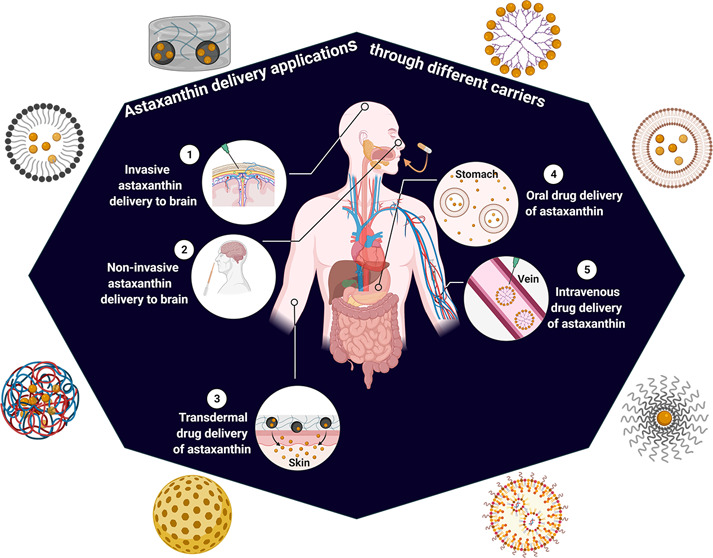

Astaxanthin (AXT) is one of the most important fat-soluble carotenoids that have abundant and diverse therapeutic applications namely in liver disease, cardiovascular disease, cancer treatment, protection of the nervous system, protection of the skin and eyes against UV radiation, and boosting the immune system. However, due to its intrinsic reactivity, it is chemically unstable, and therefore, the design and production processes for this compound need to be precisely formulated. Nanoencapsulation is widely applied to protect AXT against degradation during digestion and storage, thus improving its physicochemical properties and therapeutic effects. Nanocarriers are delivery systems with many advantages—ease of surface modification, biocompatibility, and targeted drug delivery and release. This review discusses the technological advancement in nanocarriers for the delivery of AXT through the brain, eyes, and skin, with emphasis on the benefits, limitations, and efficiency in practice.

## 1 Introduction

Astaxanthin (AXT), a highly potent xanthophyll, is a red, lipid-soluble carotenoid.^1,2^ Despite its numerous health-benefits, AXT has limited use in the pharmaceutical and food industries due to its poor solubility in water and lack of stability when exposed to oxygen, light, and high temperatures;^3,4^ conjugation with fatty acids or proteins promotes its natural stability.^5^ Notably, the oral intake of AXT is equally limited due to its low rate of dispersion in blood vessels as well as its low cellular absorption. An extensive effort has been made to boost the bioavailability, stability, and solubility of this powerful antioxidant by encapsulation. This method may protect AXT from gastric fluid and allow its gradual release in the intestinal fluids.

Among the various methods of encapsulation, liposomes, spray drying, solvent evaporation, ionic gelation, coacervation, and lyophilization are used in AXT formulation. Controlling the particle size and further purification of the product due to the use of solvents are the limitation of these encapsulation techniques. Recently, supercritical fluid precipitation is an environmentally friendly technology that has been used for the encapsulation of AXT. In a new study, supercritical carbon dioxide (SC-CO_2_) was employed in contact with the emulsion of AXT, ethyl acetate saturated water, and ethyl cellulose to encapsulate AXT. This method preserved the antioxidant activity of AXT and generated a high production capacity with an encapsulation efficiency of 84%.^6^ In another study microspheres of AXT were prepared using SC-CO_2_ technology with an encapsulation efficiency of 91.5%; AXT was dissolved in poly(l-lactic acid), dichloromethane, and acetone and then was evaporated into the bulk SC-CO_2_.^7^ The size and structure of capsules are significant factors to be taken into account for encapsulation of AXT. Structures of multiple layers (liposomes, oil-in-water emulsions) with nanometric scale provide higher stability and biological activity and allow controlled release of AXT.^8^ Not only do these micro-/nanocapsules protect AXT against gastrointestinal digestion and later release in the intestine, but also smaller AXT-loaded carriers (<500 nm) can be absorbed by endocytosis or through Peyer’s patches, thus enhancing the bioavailability of AXT.^9^ Therefore, the physicochemical properties, such as the size, charge, surface, and composition of the lipidic particles, can protect AXT against enzymatic digestion and enhance its stability and bioavailability.^10^ These nanoparticles, due to their lipophilic properties, can adhere to membranes and penetrate cells, and therefore, they have been suggested as excellent AXT carriers across the intestinal barrier. Nanostructured lipid carriers seem to be more stable to degradation than liposomes in the presence of gastric acid secretions and pancreatic lipases. For instance, the use of phospholipids, saturated lipids, or phytosterols can enhance the stability of carriers. Also, the surfactant-based delivery systems such as niosomes have resistance to hydrolysis and acid media.^11^ Other materials such as alginate/gelatin and whey protein/gum Arabic in gastric acidic pH are insoluble and prevent degradation but in intestinal pH facilitate dissolution where the encapsulated AXT is released.^6^ Therefore, the selected materials for encapsulation modulate the release of AXT in the intestine and cause resistance to its pH, hence preserving micro-/nanocapsules until degradation. Also, the delay in gastrointestinal transit of nanocapsules depends on the mucoadhesive properties of materials and the small particle size. Chitosan-based nanoparticles present advantages for loading AXT, as they are safe, biodegradable, and have high affinity to the cell membrane, thus improving the transport of AXT through the epithelial tight junctions. However, these nanoparticles are degraded under low pH conditions and cannot protect AXT during gastrointestinal digestion. Studies have demonstrated that blended chitosan with casein and oxidized dextran or other nonionic polymers enhance the physicochemical stability of these nanoparticles.^12^ One major criterion for choosing an efficient and suitable biopolymeric or lipid-based nanoencapsulation system for transportation of astaxanthin is the structure, barriers, and cellular composition of the target organ (brain, skin, and eye, etc.). The choice of an appropriate encapsulant material helps enhance the bioaccessibility, solubility, and long-term stability of astaxanthin in target organs. Overall, based on recent studies, chitosan (carbohydrate biopolymer) in combination with proteins or other carbohydrates is a valuable carrier for astaxanthin, and among lipid-based nanocarriers, nanoniosomal and nanostructured lipid vehicles are efficient systems relative to other lipid-based systems. Added parameters in the selection of a proper encapsulant, are its availability and reasonable price, and also the suitable route of its administration (oral, ocular, parenteral, etc.).^13−16^

The goal of this review is to highlight the properties and applications of AXT-encapsulated nanocarriers. In this regard, the limitations, advantages, and practicality of recent innovations and developments including nanodelivery systems of AXT for various ailments (e.g., neurological, ocular, and dermal disorders) are deliberated.

## 2 Source, Structure, and Extraction

**Figure 1:**
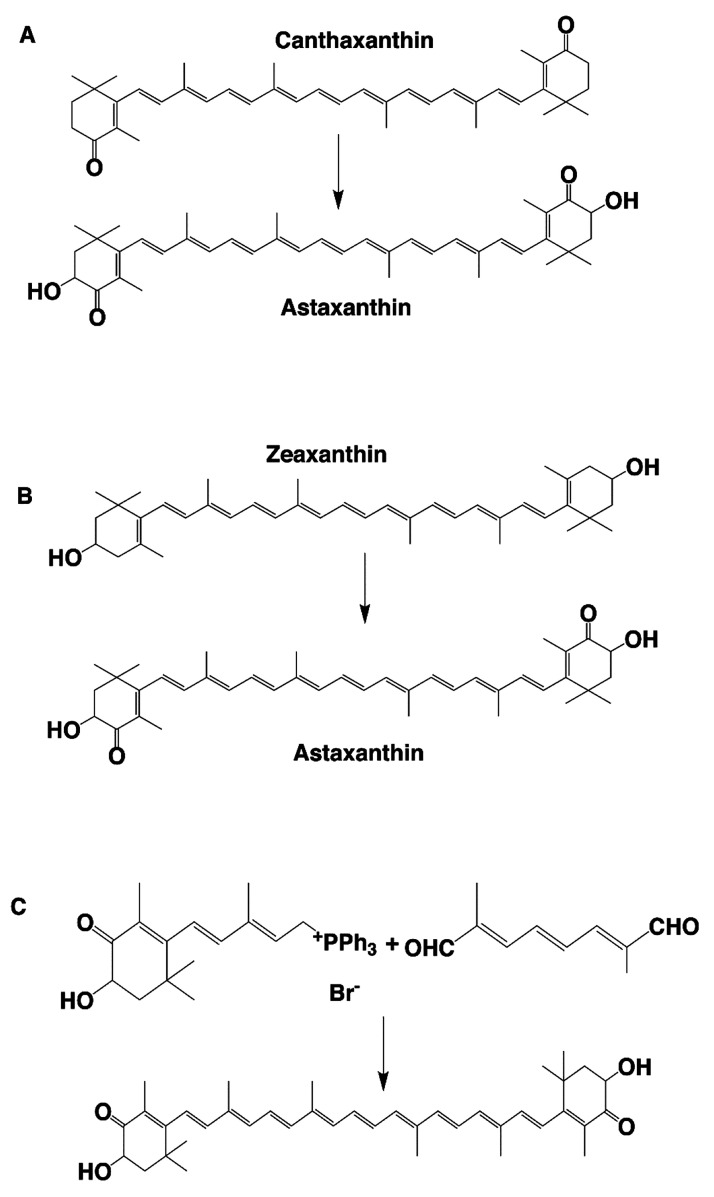
Three strategies of the chemical synthesis of AXT: (A) hydroxylation of canthaxanthin; (B) oxidation of zeaxanthin; and (C) Wittig reaction.

AXT is a xanthophyll, with the molecular formula C_40_H_52_O_4_ and molar mass 596.84 g/mol. It is naturally present in many sea creatures and living organisms, namely salmon, shrimp, krill, lobster, microorganisms, and some plants.^17^ Synthetic AXT, on the other hand, is produced by petrochemical products following a multistep process. Three different methods are used for the chemical synthesis of AXT: hydroxylation of canthaxanthin (Figure 1A), oxidation of zeaxanthin (Figure 1B), and Wittig reaction (a dialdehyde with two phosphoniums) (Figure 1C). To date, only natural AXT has been approved for human consumption. It is used as an expensive material for various therapeutic applications, whereas the use of the synthetic form falls mainly into aquaculture appliances merely as a feed additive.^18^ Notably, the antioxidant activity of natural AXT is 20–50 times stronger than that of synthetic AXT. It has exhibited better therapeutic performance and has shown no toxic effects.^19^ Therefore, the consumption of natural AXT and demand for it have grown more dramatically than those for the synthetic counterpart. Natural AXT is mainly derived from algae (*Haematococcus Pluvialis*), bacteria (*Paracoccus haeundaensis, Paracoccus carotinifaciens*), and yeast (*Phaffia rhodozyma/Xanthophyllomyces dendrorhous*). *Haematococcus pluvialis* is a freshwater microalgae and is known as a great source of natural astaxanthin.^20,21^ Many companies are producing natural AXT from algae, due to its mounting importance in the pharmaceutical industry.^22−24^ A considerable challenge in biotechnological production of AXT is the downstream processes. As AXT is produced intracellularly and high-purity AXT is needed for nutraceutical and pharmaceutical applications, high operating costs are mostly encountered; thus, the cost of downstream processes is nearly 80% of the production cost.^25,26^ An effective downstream process can reduce production costs and develop productivity.

**Figure 2:**
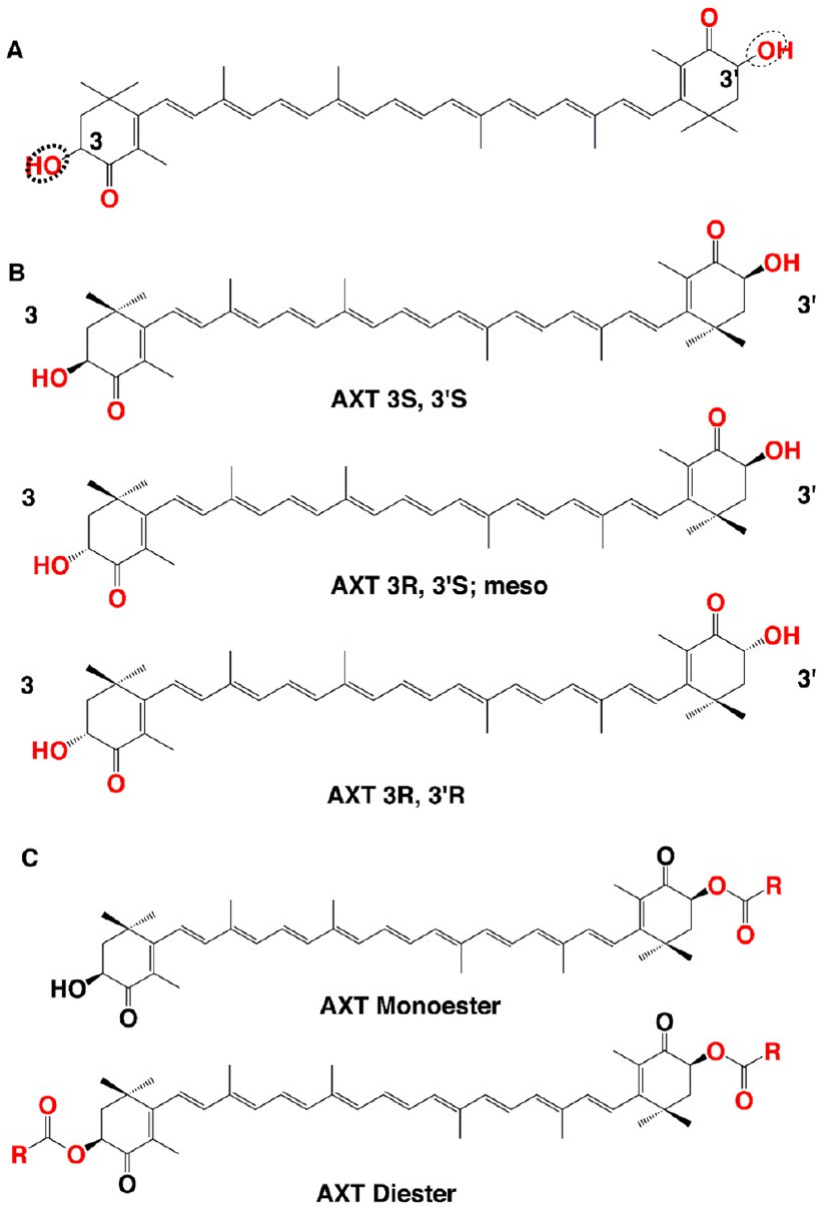
(A) Chemical structure and (B) stereoisomers of AXT. (C) Structures of AXT monoester and diester forms.

AXT consists of two terminal rings joined by a polyene chain. The molecule contains two asymmetric centers located at the 3 and 3′ positions of the β-ionone ring with a hydroxyl group (-OH) on either end of the molecule (Figure 2A). A chain of conjugated double bonds is extended at the center of the molecule which is responsible for the antioxidant activity of AXT.^27−30^ In view of the presence of oxygen in its rings, AXT possesses a more polar nature, making it a strong antioxidant as it can donate electrons and mop up free radicals. Notably, the configuration of stereogenic carbons at the 3 and 3′ positions in these rings defines AXT spatial isomers as chiral (3S, 3S′) or (3R, 3R′) or as meso (3R, 3′S), with the chiral configuration being the most abundant in nature (Figure 2B).

The presence of a hydroxyl and carbonyl (C=O) in each ionone ring explains features such as its polar nature and its ability to undergo esterification. Based on its source, AXT can exist in different forms such as optical R/S isomers, geometric isomers, and esterified or free forms.

Although the most predominant form of AXT in nature is the esterified form, the nonesterified form can also be found. AXT is found in three different forms based on its two hydroxyl groups: the nonesterified form (free form), monoesterified form (one hydroxyl group esterified with fatty acid), and diesterified form (two hydroxyl groups esterified with fatty acid) (Figure 2C). Various sources of AXT synthesis contain different ratios of these three forms. For instance, AXT extracted from yeast *Xanthophyllomyces dendrorhous* is the (3R, 3′R) isomer in the free form, while *Haematococcus pluvialis* biosynthesizes the (3S, 3′S) isomer in the monoesterified form predominantly (Table 1).^31^

**Table 1:** Comparison of the Physicochemical Properties of AXT from Different Sources

**source**	**isomers**	**3,3**′**-OH group modification**	**properties of dominant form**	**ref**
*Haematococcus pluvialis*	3S, 3′S	70% monoesterified, 25% diesterified, 5% free form	high stability	(32, 33)
*Paracoccus carotinifacience*	3S, 3′S	100% free form	unstable, sensitive to oxidation, higher bioaccessibility	(34, 35)
*Phaffia rhodozyma*	3R, 3′R	100% free form		
synthetic	1(3S, 3′S), 2(3R, 3′S), 2(3S, 3′R), 1(3R, 3′R)	free form		

The ratio of stereoisomers in synthetic and natural AXT is inherently different. Synthetic AXT contains the (1(3R, 3′R):2(3R, 3′S):1(3S, 3′S)) that is the free form, whereas variable ratios of tree stereoisomers exist in natural AXT mainly in a complex with proteins or lipids, or the esterified form. The remarkable bioactivity of AXT originates from the 3S, 3′S isomer which explains a better bioavailability after dietary supplementation with natural AXT than the synthetic form.The research conducted by Yang et al. indicated that diesterified AXT with long-chain and saturated acids has more stability than other forms of AXT. They showed that the stability of AXT directly correlated with the esterification degree, length of the carbon chain, and saturated state of the fatty acid. Furthermore, the decrease in the esterification degree, the decrease in the length of the carbon chain, and the increase in unsaturation of the fatty acid of AXT are beneficial for its bioavailability. During digestion, monoesterified AXT with short-chain and unsaturated fatty acids was easily hydrolyzed. Therefore, the bioavailability of free AXT is considerably higher than that of monoesterified AXT, and that for the monoesterified form is notably greater than that of the diesterified AXT.^33^ After supplementation with AXT (either free or esterified), the only form found in human blood is the free form. Moreover, studies in humans demonstrated that the free form of AXT is the primary active form and has more bioavailability than the esterified form.^36^ It is speculated that the amount of esterified AXT at the uptake site is limited due to the need for gastrointestinal hydrolysis of these esters before absorption.^37^ In the purification step of the downstream process, impurities such as salts, cell debris, other carotenoids, solvents, proteins, esters, and other contaminants are separated and free natural AXT (99% purity or more) is obtained. After removal of ester groups, free AXT and its isomers easily can be analyzed by chromatographic techniques; free AXT would form useful pharmaceutical antioxidants as they can be bound to water-soluble groups.^38^ Mimoun-Benarroch et al. demonstrated that the absorption of esterified natural AXT from *H. pluvialis* is slower than free AXT from *P. carotinifaciens* and *P. rhodozyma*; hydrolysis of the esterified form in the intestinal lumen before absorption probably contributes to the decrease in the uptake process. Additionally, these esterified AXTs cannot be identified by chromatographic analysis unless their fatty acid chains are removed.^39^ However, some studies claim that esterification makes AXT more soluble and enhances its stability to oxidation; therefore, it can have better pharmacological properties than free AXT.^40^ Consequently, some researchers have carried out purification of *H. pluvialis* AXT and recovered a high percentage of purified free or monoester AXT.^41,42^ Normal-phase chromatography coupled with reverse-phase chromatography can be used in separation of free and esterified AXT (mono and diesters) in 25 min.^43,44^ The antioxidant activity of various forms of natural AXT is still debated. It has been claimed that free AXT is more efficient than the esterified AXT,^45^ while some others have reported the esterified form with better antioxidant activity.^46−49^ The study of Rao et al. on a skin cancer model in rats showed that esterified AXT has better antioxidant and anticancer potency than the free form.^50^ Also, comparing these two forms on exercise performance in mice exhibited that esterified AXT significantly promoted muscular endurance, protected erythrocytes from oxidative damage, and increased the running time.^51^

**Figure 3:**
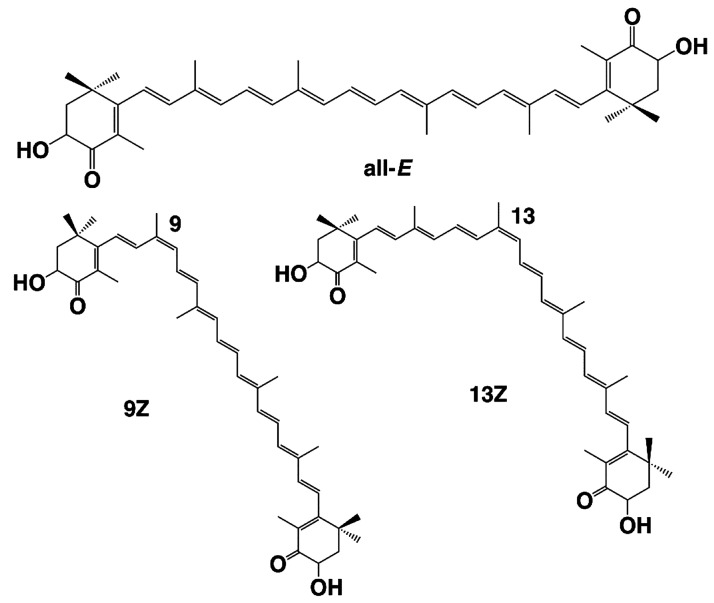
Structures of *E*/*Z* isomers of AXT.

In view of the presence of several conjugated double bonds, two kinds of geometrical isomerization occur in the AXT molecule: *Z* and all-*E* isomers (Figure 3). The most representative AXT in nature is the all-*E* stable isomer when the carbons are located in the *E* positions at double bonds. Less stable but more beneficial *Z* isomers (a mixture of the 9*Z* and 13*Z* isomers) are obtained when AXT extracts are affected by factors such as the metal ions,^52^ solvents, heat, or pH of the reaction medium.^53^ Viazau et al. examined the isomerization of AXT under heat and overlighted conditions and in both in vitro and in vivo (*H. pluvialis* cells) systems. In the first 5 h of light treatment in the in vitro conditions and in the presence of methanol, both *Z*-isomers increased to 5% and then decreased, but during the whole period of heat treatment, the amount of accumulated *Z*-isomers was increased. In *H. pluvialis* cells, under conditions of intense light and sodium acetate, the accumulation of *Z*-isomers at first reached 45% and then decreased; reduction of isomers may be due to de novo synthesis of all-E-AXT and the oxidative degradation of AXT. To increase the total production of AXT in *H. pluvialis* cells, the presence of sodium acetate and long-term light is necessary, and to increase the production of *Z*-isomers, only short-term light is sufficient.^54^ Several studies have investigated the beneficial features of Z isomers relative to *E* isomers of AXT. Yang et al. have noted the selective accumulation of 13*Z*- AXT in human plasma with the assertion that *Z* isomers are more fruitful for human health.^55^ As the *Z* isomers are more soluble in organic solvents, their extraction is more efficient when *Z*-isomerization accelerating catalysts are added to the extraction solvent; so, they have better extractability than the all-*E* isomer.^53^ As a result of some alternations taking place in the physicochemical properties of AXT in the *Z* configuration, as they change from a crystalline state to an amorphous (oily) state, processes such as extraction, emulsification, and micronization are facilitated by safe and sustainable solvents.^56^ Higher dispersibility and solubility of AXT -Z isomers lead to higher bioaccessibility and bioavailability of this molecule; 13*Z*- AXT has higher bioaccessibility than 9*Z*- and all-*E*-AXT in the *in vitro*-digestion model.^55^*Z-*isomerization also effects the anticancer, antioxidant, anti-inflammatory, antiaging, and antiatherosclerotic activities of AXT.^56^ Yang et al. demonstrated higher inhibition of inflammation for *Z*-isomers, especially 9*Z*, by decreasing the expression of NK-κ, IL-8, TNF-α, and COX2 in the Caco-2 cell monolayer model.^57^ Better antiaging activity of 9Z- AXT was observed when the median life span of *Caenorhabditis elegans*, fed with it, increased by 59.39% compared to an increase by 30.43% when fed by all-*E*-isomers.^58^ All these changes in the function and activity of AXT-*Z* isomers are due to the altered physicochemical characteristics of this molecule. Some physicochemical properties influencing the *E/Z*-isomerization are the solubility, color value, stability, crystallinity, and melting point. Changes in the Gibbs free energy affect the stability of the *Z*-isomer, which in turn affects its antioxidant properties.^59^ Liu and Osawa have shown the robust antioxidant effects of the *Z*-isomer (especially 9*Z*-AXT) in highly efficient radical scavenging activity and also suppressing the production of ROS in neuroblastoma cells as well as the inhibition of induction of hydroperoxides.^60^ On the other hand, Yang et al., by different antioxidant activity assays, showed that 13*Z*- AXT has stronger antioxidant activity relative to all-*E* and 9*Z.*^61^*Z*-Isomers have a higher solubility in organic solvents, vegetable oil, and SC-CO_2_ which enhances their bioaccessibility. Likewise, the uptake of *Z*-isomers into bile acids improves, and their internalization to the Caco-2 cells by carotenoid transport proteins is more efficient (Table 2).^55^

**Table 2:** Propertes of Different Geometric Isomers of AXT^a^

**property**	**type of isomer**	**type of assay**	**ref**
antioxidant capacity	13*Z* > all-*E* > 9*Z*	CAA assays (Caco2-BBe1/HT-29)	(61)
	13*Z* > 9*Z* > all-*E*	ORAC-L, PLC assays	(61)
	9*Z* > 13*Z* > all-*E*	DPPH and lipid peroxidation assay (SH-SY5Y cells)	(62)
transport efficiency	9*Z* > 13*Z* > all-*E*	Caco-2 cell monolayer model	(55)
bioavailability/bioaccessibility	*Z*-isomers> all-*E*	oral-dosing test (human)	(63)
	all-*E* > 13*Z* > 9*Z*	oral-dosing test (rainbow trout)	(64)
	13*Z* > 9*Z*, all-*E*	oral-dosing test (human)	(65)
	13*Z* > 9*Z*> all-*E*	digestion model (Caco-2 cells)	(55)
stability	all-*E* > 9*Z* > 13*Z*	storage tests (heating and filtration)	(66)
	all-*E*, 13*Z* > 9*Z*	pH test	(61)
solubility	*Z*-isomers > all-*E*	organic solvents	(53)

a Abbreviations: ORAC-L assay, oxygen radical absorbing capacity assay for lipophilic compounds; PCL assay, photochemiluminescence assay; CAA assay, cellular antioxidant activity assay; DPPH, 2,2-diphenyl-1-picrylhydrazyl; bioaccessibility, the amount of AXT available for absorption in the gut after the digestion process; bioavailability, the amount of AXT which reaches the site of physiological activity after administration.^25^

**Figure 4:**
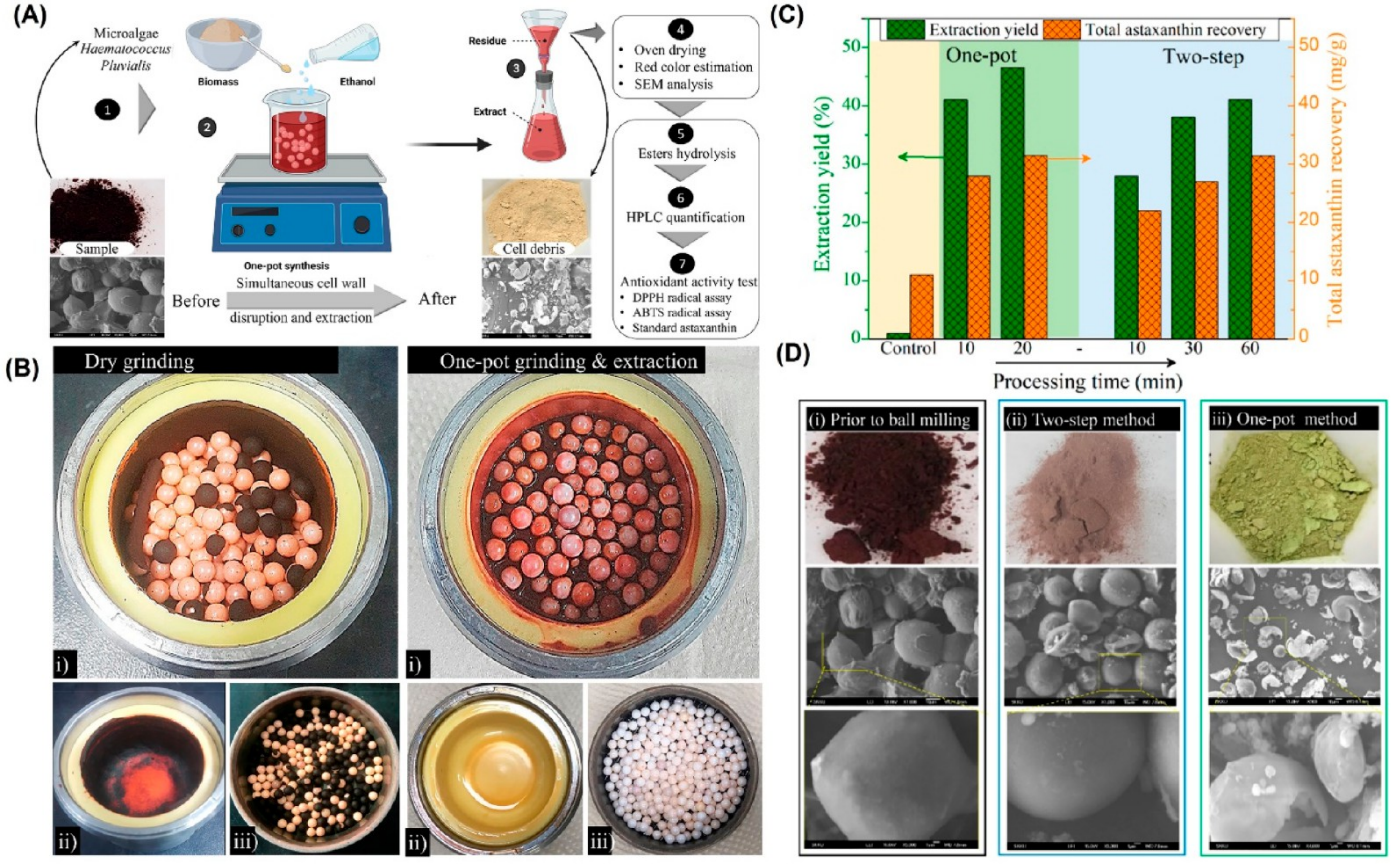
(A) Schematic indicating the one-pot strategy for extraction of AXT from *Haematococcus pluvialis*. (B) Comparison between dry and wet techniques by digital camera images: (i) right after the ball milling process, (ii) of the containers wall, and (iii) of the zirconia balls after the process. (C) AXT yield for the control (freeze-dried *Haematococcus pluvialis* through Soxhlet extraction by acetone without applying ball milling (12 h)), one-pot strategy (up to 20 min), and two-step technique (up to 60 min). (D) Digital camera and SEM images showing the cell debris (i) prior to ball milling, (ii) after the two-step method, and (iii) after the one-pot method. Reprinted with modification from ref (67) with permission from American Chemical Society.

An effective downstream process reduces production costs and develops productivity. Not surprisingly, the natural AXT obtained from *Haematococcus pluvialis* is expensive and has only 1% of the total AXT market share while the rest goes to the synthetic counterpart.^67^ However, there are emerging strategies which have the potential to increase the natural AXT’s share in the market. It is known that AXT can be concentrated in *Haematococcus pluvialis* up to 5 wt % of its dry weight at the aplanospore stage under undesirable conditions among which high salinity, high temperature, and more light can be enumerated. On the other hand, if undesirable conditions prevail, it would culminate in the accumulation of AXT; the increase in the AXT is accompanied by the formation of a acetolysis-resistant wall around the cells with a thickness up to 2.3 μm, an impediment for the extraction process.^68,69^ Only 5% of AXT in the cells is in the free form and the rest is bound to fatty acids. The extraction of the free form plus its derivatives requires the rupture of the cell wall, but preserving the AXT bioactivity during the process is of vital importance, making it a remarkable challenge in the field.^70^ A mild one-step strategy has been reported to yield 47 wt % through the recovery of AXT from the mature cysts of *Haematococcus pluvialis*. In this method, the cell wall of the cyst cells is completely ruptured under mild conditions (200 rpm, room temperature, and atmospheric pressure) in a short time (≤30 min); ensuing extracts are realized using different solvents generally recognized as safe (GRAS), e.g., ethanol, acetone, *n*-hexane, ethyl acetate, and isopropyl alcohol). Astaxanthin recovery is the highest in ethanol, followed by that in acetone, ethyl acetate, isopropyl alcohol (IPA), and hexane. Figure 4 exhibits the optimized one-pot process together with the usual dry grinding and two-step process to make a comparison. The pretreatment to rupture the cells wall is avoided in the one-step strategy, making the process efficient relative to the previous studies.^71,72^ The difference between dry and wet methods is discernible in Figure 4B; the dry ball milling, which is adopted widely, causes the formation of cells debris on the balls and the chamber’s wall followed by their aggregation and, hence, is less efficient. In the case of the two-step process, an initial grinding is performed followed by the extraction via Soxhlet, supercritical fluid, or other means with a low yield, while the one-pot process allows the AXT extraction in high yield in a short time at ambient temperature.^67^

**Figure 5:**
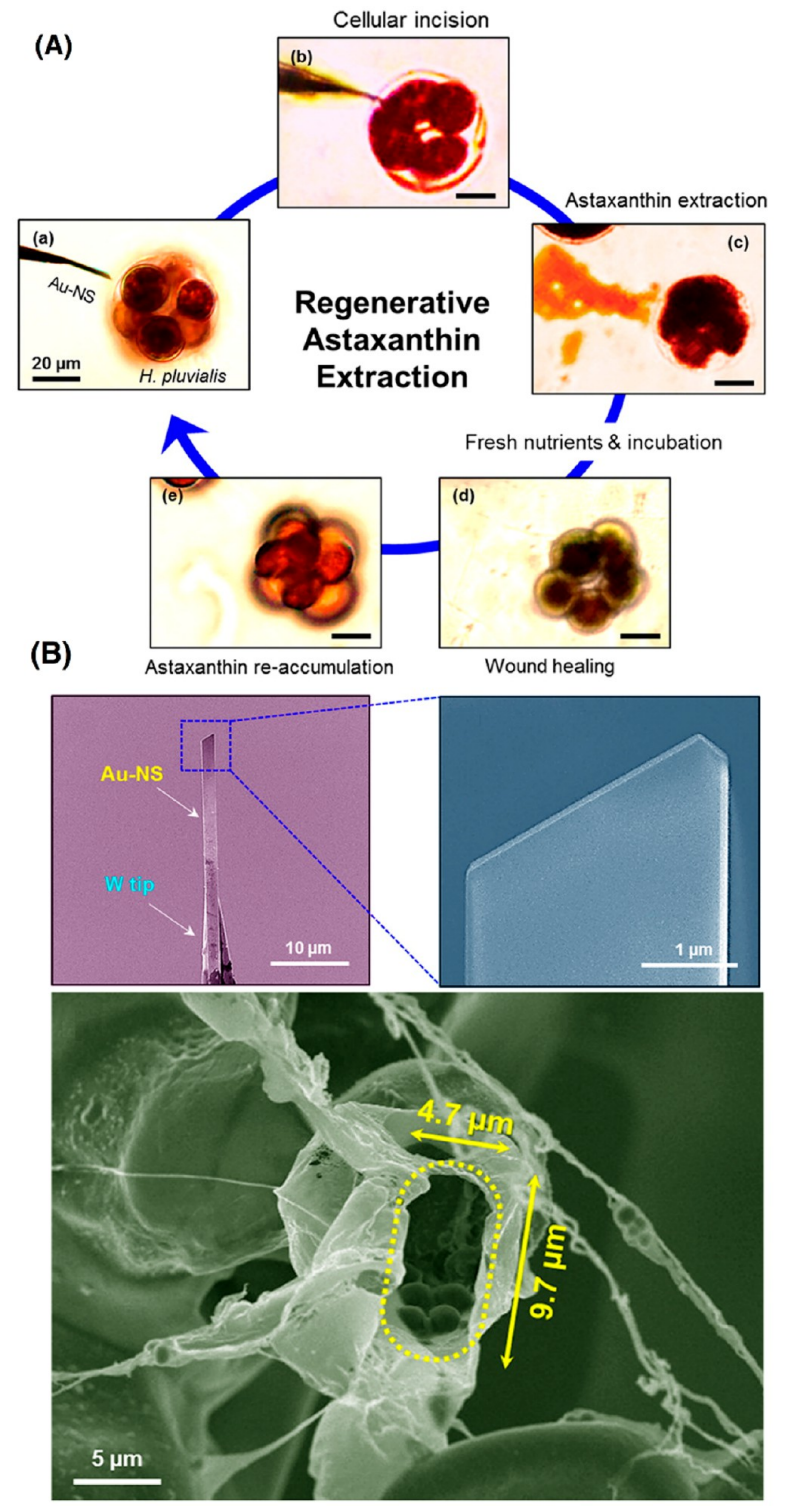
(A) Regenerative AXT extraction from *Haematococcus pluvialis* through the gold manipulator. (B) SEM micrographs of the gold manipulator and incised cell. Reprinted from ref (74) with permission from American Chemical Society.

Although the one-step strategy afforded the highest amount of AXT from *Haematococcus pluvialis*, it is considered an invasive approach as it entails complete disruption of the algae. The biorefinery of microalgae comprised some steps such as cultivation, harvesting, and subsequent extraction, which is a costly and time-consuming endeavor. There is a noninvasive strategy that is capable of reducing both the time and cost-termed microalgae milking.^73^ The same as milking cows, the idea behind this process is to reuse the biomass for a prolonged production; an innovative strategy has been adopted to extract AXT multiple times from a single *Haematococcus pluvialis* cell. The process begins with an incision in the cell wall through a gold nanoscalpel followed by extraction of AXT and finally wound healing by providing incubation and nutrients. Importantly, the extraction is synchronized with chlorophyll leakage besides AXT. After the extraction process, the nutrient addition stopped leaking the pigments and the chlorophyll content increased again, which is vital for preserving the cellular metabolism. The relationship between chlorophyll and AXT is found to be inverse; enhancment in the AXT content up to twice that of the control groups was discerned after the first extraction process^74^ (Figure 5). Of course, more research is required to optimize the milking process, and it is worth researching as the process is reusable multiple times as desired.

Xanthophyll carotenoids, to which AXT belongs, are solubilized in the small intestine after ingestion. This process is carried out in mixed micelles which contain bile acids, phospholipids, cholesterol, and fatty acids. Then, these carotenoids enter the epithelial cells by a simple and facilitated diffusion through their cytoplasmic membranes. Once they are broken up, carotenoids are stored in the liver. They are next resecreted as very low-density lipoproteins, low-density lipoproteins, and high-density lipoproteins into the blood and transported to the tissues. The polar ends of AXT make it more readily absorbable than other nonpolar carotenoids such as lycopene. It has been shown that esterified AXT is hydrolyzed (fatty acids removed from the either ring) before being transported as low-density lipoproteins.^75,76^ AXT is similar in structure to the β-carotene, with the former having 13 conjugated double bonds, whereas the latter has 11; the ability of carotenoids to neutralize free radicals enhances with increasing conjugated double bonds and the presence of a functional group in its terminal rings.^77^ Polar AXT spans the membrane, with its polar end groups extending toward the head regions of the membrane bilayer. As a result, AXT stops free radical chain reactions and scavenges lipid peroxyl radicals and ROS (endogenous ROS) on the membrane surface, while its polyene chain can trap ROS in the interior of the membrane.^78^ The toxicity and efficacy of soft capsules of oil-based AXT have been evaluated by Satoh et al. According to this analysis, no safety issues have been observed while the metabolic syndromes were improved. The United States Food and Drug Administration and the European Food Safety Authority have approved AXT as a dietary supplement, a food ingredient, and an additive. Until now, the AXT extracted from *H. Pluvialis* and *P. carotinifaciens* has been authorized for human consumption at dosages ranging from 12 to 24 mg and 6 mg per day, respectively, for up to 30 days.^79,80^

## 3 AXT Function in the Human Body: Antioxidant Activity and Signaling Pathways

AXT has exhibited prooxidant properties. It is known that the low ROS amounts are advantageous for gene expression, cellular signaling, and the stimulus of antioxidative defense mechanisms.^81^ Several studies have demonstrated that AXT is more potent than beta-carotene in scavenging free radicals induced by internal (inflammation, aging, stress, and cancer, among others) or external sources (cigarette smoke, pollutants, UV radiation, etc.)^82,83^ and conserves unsaturated fatty acid methyl esters by preventing peroxidation. Besides, AXT esters have shown high antilipid peroxidation activity.^84,85^ The health-promoting impact of AXT on many diseases has been demonstrated in several studies wherein the promising therapeutic effects of AXT were highlighted.^86,87^ AXT strengthens and modulates the immune system and increases antibody production in a T helper-dependent manner. Thus, it raises the number of antibody secretory cells from spleen cells and the production of immunoglobulins by blood cells.^88,89^ Its very strong antioxidant activity may have protective impacts on the cardiovascular system.^90^ Coombes et al. demonstrated that AXT has no effect on enhanced inflammation, oxidative stress, and arterial stiffness in renal transplant recipients.^91^ Other studies have suggested that AXT has immense effects on cardiac function, buildup joint strength, exercise performance, and postexercise recovery.^92^ Also in heart failure patients, three month consumption of AXT has an antioxidative stress effect and improves exercise tolerance and cardiac contractility.^93^ The antitumor effects of AXT including anti-inflammation,^94^ antiproliferation,^95^ antioxidation,^96^ and increasing apoptosis^95^ have been confirmed in many *in vivo* and *in vitro* studies. It also improves the functioning of the brain and can reduce or prevent brain diseases, such as Parkinson’s disease, autism, and Alzheimer’s disease.^97,98^ AXT reduces wrinkles on the skin and prevents age spots, improves skin’s elasticity, and reduces ultraviolet damage due to sun rays, hence acting as an internal sunscreen.^99,100^

AXT has a significant role on the signaling pathways of inflammation, oxidative stress, and reactive oxygen-dependent apoptosis by interrupting their signaling pathways in neurodegeneration and ocular and skin-related damage.^101,102^ Though ROS have a significant role in neuronal signaling and function, unwarranted generation of ROS is lethal for neural cell function, with permanent oxidation. AXT showed neuroprotective effects by reducing intracellular ROS and preventing mitochondrial H_2_O_2_ generation.^103^

**Figure 6:**
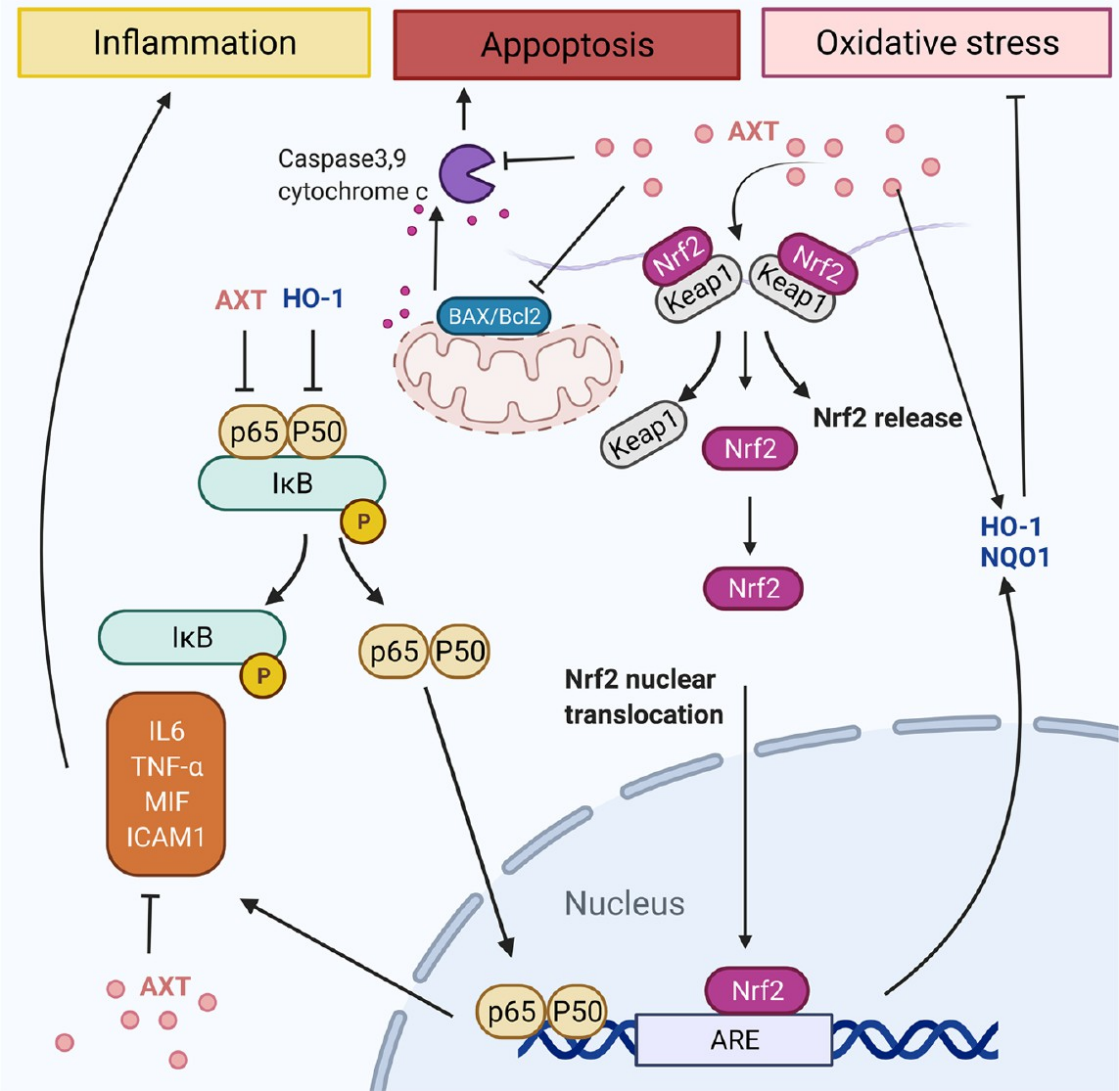
Schematic illustration of AXT’s role on signaling pathways of inflammation, oxidative stress, and apoptosis by interrupting their signaling pathways.

AXT can prevent inflammation by inhibiting the release of interleukins (ILs), tumor necrosis factor-alpha (TNF-α), and intercellular adhesion molecule 1 (ICAM1) as shown in Figure 6.^101,104^ The anti-inflammatory properties of AXT were due to its inhibition of the TLR4 pathway beyond TLR4/MyD88/NF-κB pathway regulation,^105^ downregulation of TLR4 and MyD88 expression, and inhibition of TLR4/MyD88/NF-κB pathway activation, which has a considerable role in regulating burn-induced renal tissue inflammation.^106^ AXT has ocular anti-inflammatory assets by impeding the NF-kB signaling pathway over suppression of TNF-α, NO, and PGE2 generation.^107^ Moreover, AXT suppressed the choroidal neovascularization by downregulation of ICAM-1, macrophage-derived VEGF, MCP-1, and IL-6 as inflammatory mediators.^108^ Also, it can effectively support additional tissue protection by maintaining the oxidant/antioxidant balance associated with its unique structure.^109^

To block the oxidative stress, AXT activates Nrf2/antioxidant response elements (Nrf2/ARE), inhibits the phosphorylated extracellular regulated protein kinase/extracellular regulated protein kinase ratio (p-ERK/ERK), and increases the release of NAD(P)H quinine oxidoreductase-1 (NQO-1) and heme oxygenase-1 (HO-1). It has been shown the Kelch-like ECH-associated protein 1 (Keap1)-Nrf2-ARE has a critical function in the antioxidant response of cells.^101^ AXT antioxidant mechanisms additionally include regulating the PI3K/Akt signaling pathway.^110^ AXT acting as a shield for photoreceptor cells from oxidative stress reduced apoptosis due to stimulation of the PI3K/Akt/Nrf2 signaling pathway at hyperglycemia conditions. AXT diminished the retinal ganglion cells and Muller cell damage via enhanced HO-1 production. Various signaling pathways are incorporated for increasing the cellular resistance toward oxidative stress. In this way, the Nrf2-ARE pathway plays an essential role and maintains cell function (Figure 6).^101,111^ One transcription factor attached to the ARE is Nrf2, which encourages Phase II enzyme expression. The interaction of Nrf2 with chaperone Keap1 occurs at the lack of oxidative damage. Contrariwise, in oxidant conditions, Nrf2, detached from Keap1, as its activated form, and translocated to the nucleus, attaches to the ARE and stimulates Phase II enzyme expression, for instance, heme oxygenase-1 (HO-1) and NQO1.^81,104^

AXT shows prooxidant properties and can create trace quantities of ROS instead of quenching them, which activates the expression of HO-1 and adjusts the GSH-Px expression and activity via the ERK-Nrf-2/HO-1 signaling pathway.^81^ This generated ROS was innocuous to the cells because pristine AXT endorsed proliferation of cells and improved the activity of GSH-Px and SOD enzyme and showed protective effects against H_2_O_2_-induced oxidative stress in HUVECs and reduced the ROS production induced by H_2_O_2_.^81^

Additionally, activation of Nrf2 can support the survival of retinal pericyte. AXT can activate the Nrf2-ARE pathway, thus enhancing the HO-1 and NQO1 expression and decreasing oxidative damage with protective effects from elevated glucose-induced apoptosis in photoreceptor cells (Figure 6).^124^

AXT’s role against apoptosis was ascertained by blocking caspase3,9 as shown in Figure 6, as well as cytochrome c, p-ERK/ERK, and the Bax/Bcl2 ratio.^101,111^ AXT has therapeutic effects in ischemia-reperfusion injury of the spinal cord and induced oxidative stress and neural apoptosis by PI3K/Akt/GSK-3β signaling pathway activation.^112^ The PI3K/Akt/GSK-3β signaling pathway showed neuroprotective function by inhibiting apoptosis and stimulating proliferation of the cell.^113^

## 4 Brain Delivery of AXT as a Neurological Drug-Therapy Agent

### 4.1 Blood–Brain Barrier, Anatomy, and Delivey Systems

**Figure 7:**
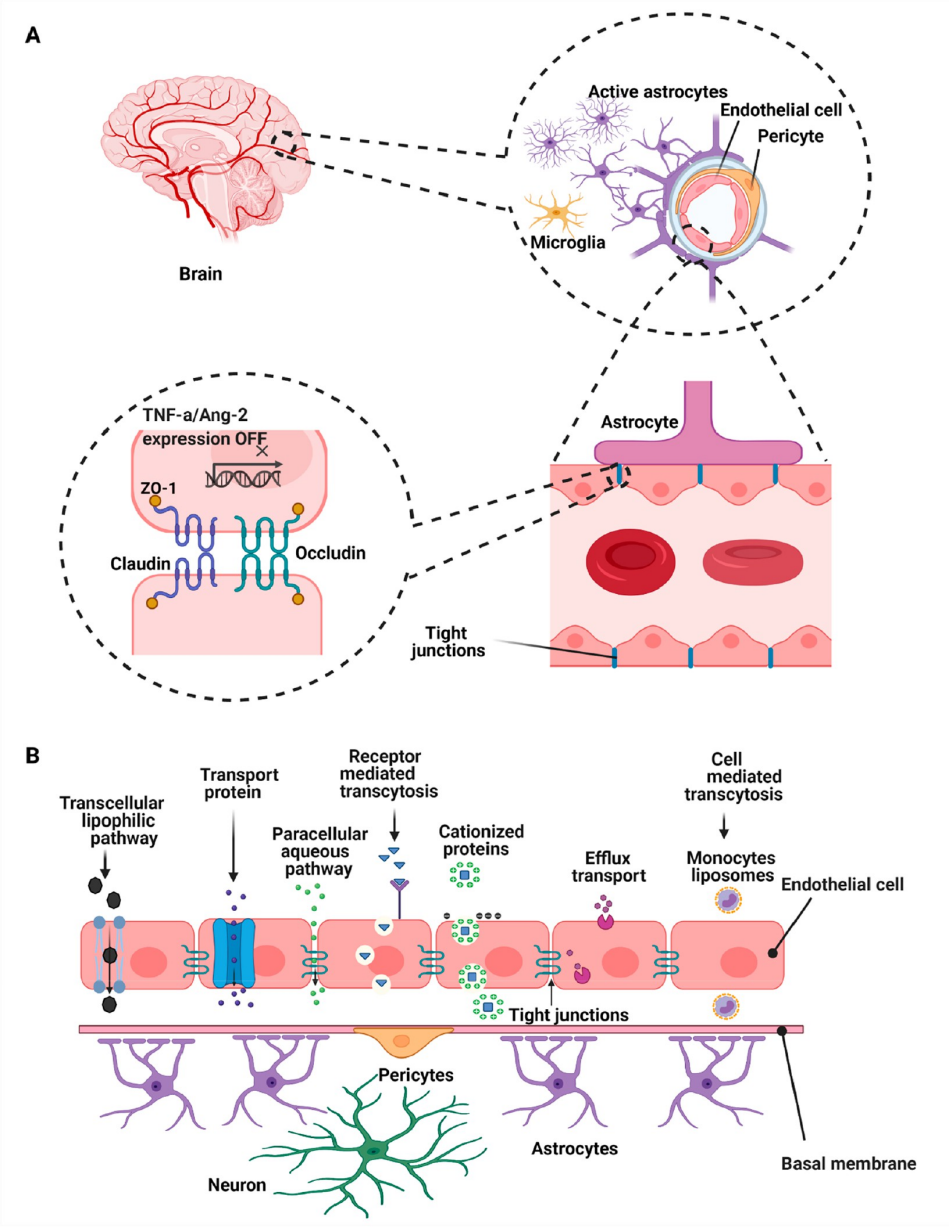
(A) Brain endothelial cells form the cellular barrier and are connected continuously by the means of tight junctions; tight junctions are the main structures of the blood–brain barrier and selectively transfer nutrients between the blood and the brain. The role of the pericytes is controlling the cerebral blood flow while astrocyte end feet are responsible for biochemical support of the endothelial cells.^122^ (B) Various strategies for diffusion through the blood–brain barrier.^127^

The central nervous system (CNS) contains the brain and spinal cord, with the latter being located inside the spine. It is separated from other parts of the human body through the blood–brain barrier, which is the boundary between the extracellular fluid of the brain in the CNS and the circulatory blood flow in the body (Figure 7A).^114^ This barrier is made up of specialized capillaries that, unlike the normal structure in capillaries, do not have the usual pores and have a tight intercellular connection. Thus, many molecules cannot pass through them through diffusion and reach the cerebrospinal fluid in the brain.^115−118^ The endothelial surface of these capillaries is covered with special proteins that allow glucose to enter the brain as well as the exchange of gas between the circulating blood and the brain from the barrier.^119^ This barrier results from tight junctions between endothelial cells in the CNS artery and restricts the passage of solutes and substances.^120^ The CNS is capable of activating the immune system in response to several forms of injury including trauma, infection, stroke, and neurotoxins.

Neuronal inflammation ensues for a variety of reasons, including infection, concussion, toxic metabolites, deformed proteins, and autoimmunity. Microglia (innate immune cells in the CNS) are activated in response to these factors and initiate the inflammatory process in nerve tissues. Although this response is initiated to protect nerve tissue against infection, it can lead to damage of nerve cells with the occurrence of neurological diseases, if the response is severe and not well controlled.^121^

Many drugs cannot penetrate through brain cells and thus preclude a therapeutic effect in brain-based diseases. Thus, the following promising strategies have been introduced for drug delivery to the brain.

(1) Transient permeability enhancement in the blood–brain barrier: Disconnection of tight junctions between endothelial cells using ultrasound/microbubbles and osmotic pressure changes, but this method allows uncontrolled entry of nanoparticles into the cell which disrupts the brain’s homeostatic function, causing brain toxicity.^123^
(2) Diffusion of small lipophilic molecules (<400 Da) through endothelial cells in two forms: paracellular and transcellular.^124^ Tight junctions hinder the diffusion of hydrophilic or lipid insoluble molecules via paracellular transport. Due to the lipid nature of liposomes and deformable liposomes (solid lipid nanoparticles (SLNs), nanostructured lipid carriers (NLCs)), it is possible for them to pass through the phospholipid bilayer of the BBB endothelial cell membrane by lipid-mediated free diffusion (facilitated diffusion) or lipid-mediated endocytosis.
(3) The transcytosis pathway through absorption, receptors, and various carriers (Figure 7B). In absorptive transcytosis, transfer begins by creating an electrostatic interaction between a positively charged particle and a negatively charged plasma membrane. This pathway is not specific to the brain and is also found in the liver, kidneys, or lungs. In one study, nanoparticles have been prepared using a polylactide polymer bound to a PEG polymer, and the results showed successful adsorption of the generated nanoparticles; the presence of PEG is intended to improve the performance of the formulation and increases the shelf life of the nanoparticles.^125,126^

**Figure 8:**
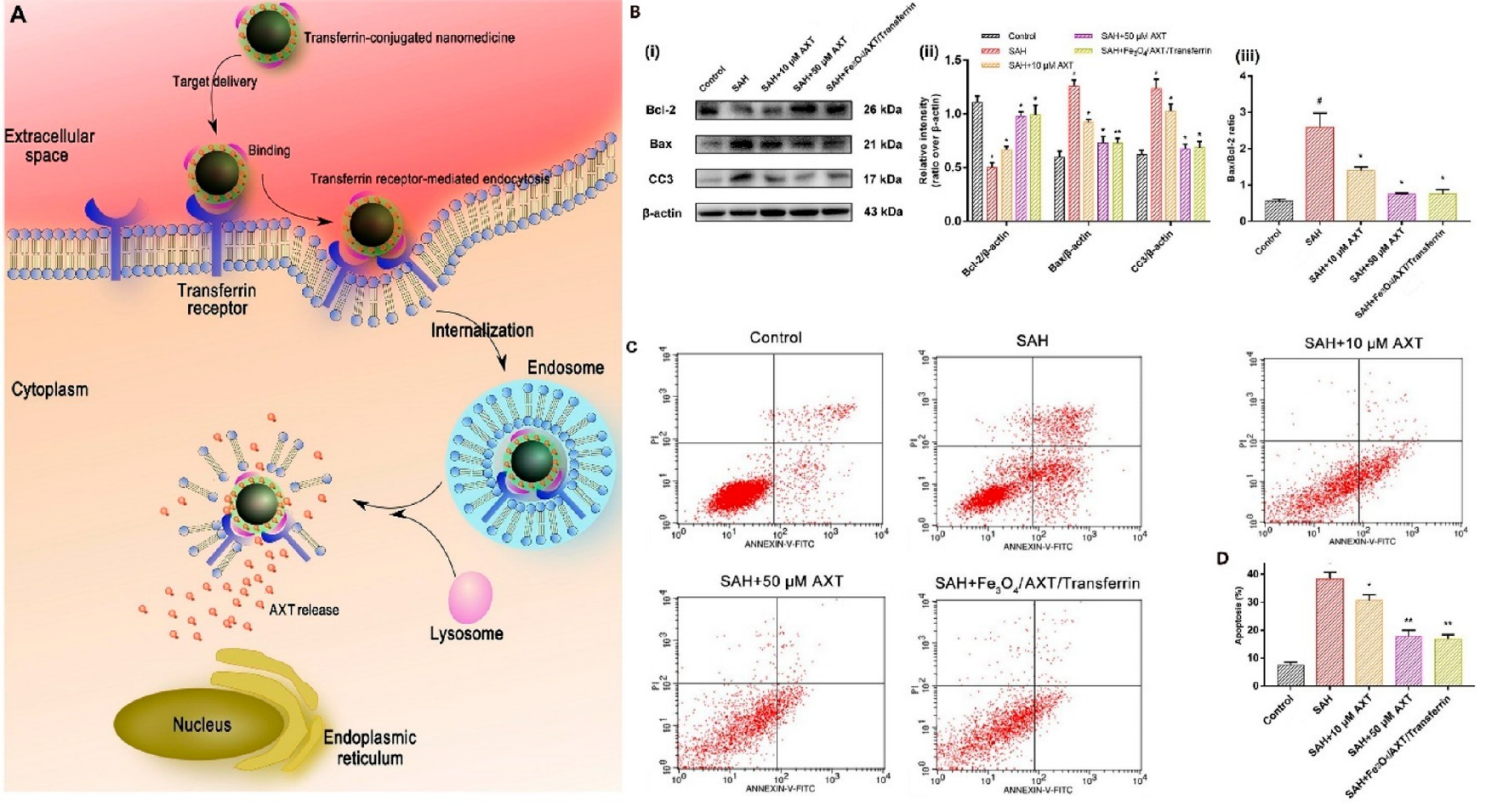
(A) Schematic of the entry mechanism of transferrin-modified and -nonmodified nanoparticles to neurons through receptor-mediation followed by the degradation of nanoparticles and AXT release. (B) Assessment of neural damage after exposure to oxyhemoglobin for pure AXT and the transferrin-modified AXT-loaded nanoparticles as follows: (i) Western blots, (ii) relative intensity analysis of Bax/ß-actin, Bcl-2/ß-actin, and cleaved caspase-3 (CC3)/ß-actin, and (iii) Bax/Bcl-2 ratio for different samples. (C) Cell apoptosis results after oxyhemoglobin exposure. (D) Apoptotic ratio of cells related to each group. #*p* < 0.05 vs control group; *p* < 0.05 vs subarachnoid hemorrhage (SAH) group; *p* < 0.01 vs SAH group. Reprinted from ref (131) with permission from Frontiers.

In receptor-mediated transcytosis, various ligands are placed on the surface of a nanoparticle that binds to cell surface receptors and is endocytosed by the cell, with receptors and transporters being used as targets, including GLUT1, LfR, and TfR.^128,129^ One of the most effective techniques is the use of transferrin, which is highly expressed on the blood–brain barrier and facilitates the nanoparticle’s penetration through the barrier.^130^ A recent study took advantage of transferrin to facilitate the penetration of Fe_3_O_4_-polyethylene glycol-encapsulated AXT nanoparticles through the blood–brain barrier for subarachnoid hemorrhage treatment. Transferrin ligand is comprised of two domains, one of which is α helixes and the other of which is ß sheets, and this ligand has high affinity toward its receptor. The cellular uptake of transferrin-conjugated nanoparticles through primary cortical neurons is significantly better than the nonmodified nanoparticles. Moreover, after exposure to oxyhemoglobin, which provides ROS, the neuronal survival gets improved and the apoptosis markers are reduced because of the AXT release.^131^Figure 8 illustrates the efficiency of transferrin-modified and -nonmodified nanoparticles for subarachnoid hemorrhage.

**Figure 9:**
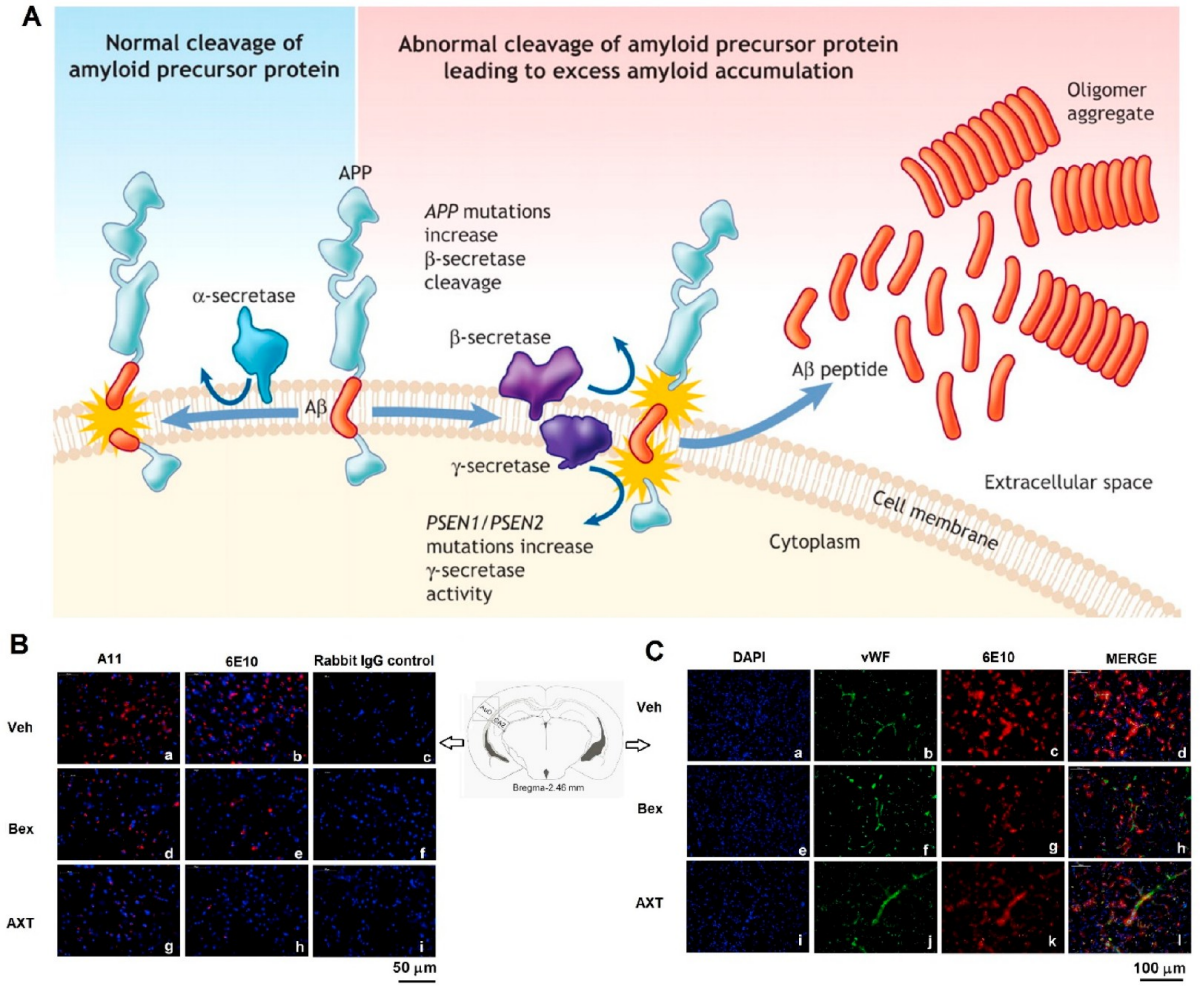
(A) As a transmembrane protein, the amyloid precursor protein (APP) undergoes a series of proteolytic cleavages by secretase enzymes. It is not amyloidogenic if APP is cleaved through α-secretase in the middle of Aβ, but the cleavage through β- and γ-secretase enzymes is accompanied by the release of neurotoxic Aβ peptides which can accumulate into an oligomer aggregate. The APP gene mutations prevent the cleavage through α-secretase followed by enabling the preferential cleavage through β-secretase. Mutations in the presenilin-1 and presenilin-2 genes (PSEN1 and PSEN2), which are regarded as the components of the γ-secretase complex, raise the cleavage through γ-secretase at this site. Notably, both situations result in the production of excess Aβ peptide. Over time, the oxidative stress causes neuronal death followed by the development of neuritic plaques typical of Alzheimer’s disease. Reprinted from ref (158) with permission from CMAJ. (B) Immunofluorescence staining was conducted on 18 μm sections of the mouse brain. (C) Immunofluorescence double staining was conducted on 18 μm sections of the mouse brains. Vehicle (Veh), bexarotene (Bex), and astaxanthin (AXT). Reprinted from ref (141) with permission of Elsevier.

**Figure 10:**
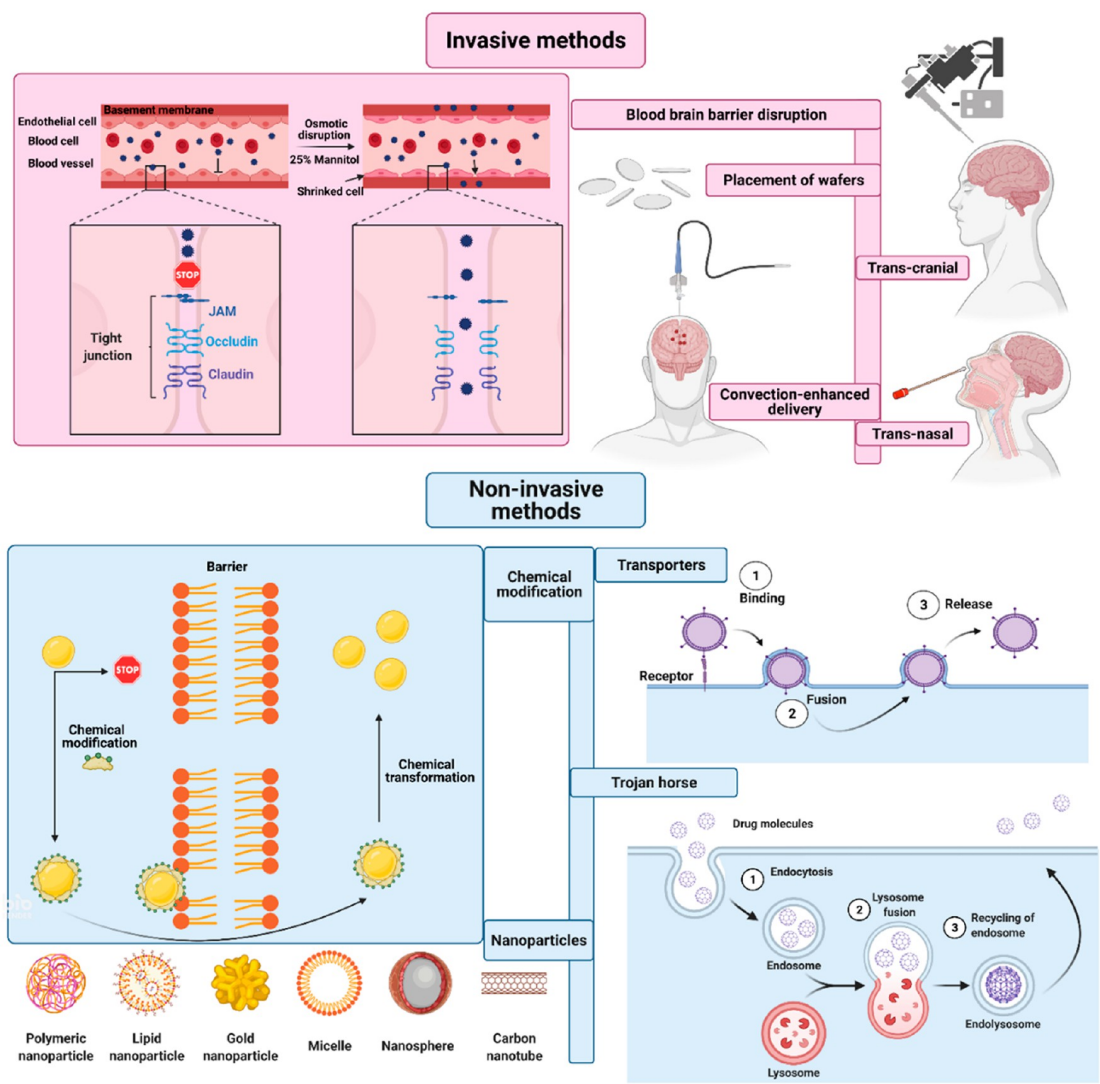
Schematic illustration showing invasive and noninvasive approaches used for drug delivery into the brain.^127^

Various strategies have been developed to increase the permeability of drugs through the blood–brain barrier.^132−134^ There are mainly two types of drug delivery to the brain, one of which is invasive and the other of which is noninvasive. Invasive methods, such as intracerebroventricular injection, osmotic and ultrasound disruption of the blood–brain barrier, and convection-enhanced delivery, help to deliver the drug directly to the desired location in the brain. Using the intracerebroventricular injection method, the drug is injected directly into the cerebrospinal fluid.^135−137^ The convection-enhanced delivery method is used to facilitate targeted drug delivery to brain tumors. In this procedure, a small hole is made in the patient’s skull to set one or more thin tubes (cannulas) to the tumor site from different angles. Then the drug is pumped into the tumor through a cannula. In ultrasound technology, the microscopic bubbles are injected into the bloodstream. Using an MRI scan, the injection is given exactly in a specific area of the brain. Then the ultrasound is transmitted to the same point through a cap placed on the head. These waves vibrate the bubbles, helping to open the tight junctions slightly, and allow the drugs to enter the brain through the created pathway.^138−140^ Also, different mechanisms that include Aβ deposition in cerebrovascular cells (Figure 9) could increase the AXT-efficiency to reduce the side effects of some drugs as well as increase the expression of some types of necessary genes in the brain.^141^ Osmotic disruption is an invasive route by which hypertonic fluids cause shrinkage of the endothelial cells of the cerebrovascular artery followed by the disruption of tight junctions of the blood–brain barrier (Figure 10).^127,142^ Another route of drug administration to the brain is by inhalation, but due to the limited absorption level of the olfactory lips, inappropriate amounts of drug molecules may reach the target;^117,143,144^ the success rate of drug delivery through these methods has been found to be inefficient.^145,146^ Opening tight connections with osmotic pressure can cause generation of toxins and other unwanted substances to enter the brain in addition to drugs. For this reason, more research has moved toward noninvasive methods. By increasing the lipophilicity of small drug molecules, the possibility of their transfer into the brain increases. As lipophilicity is enhanced, the metabolism and distribution of the drug in the body also increases, which in turn increases the dose of the drug, thus enhancing the side effects.^147−149^ Large molecules, such as peptides, proteins, or genes, are unable to cross the blood–brain barrier. In addition, these compounds have little stability in the environment, so they are rapidly metabolized and are not released into the brain. Moreover, many drugs, which have optimized molecular weight and lipophilic properties, pass through the blood–brain barrier naturally and easily, but they are quickly returned to the bloodstream by very strong outward pumps.^147^ The use of nanotechnology to enhance drug delivery to the brain without damaging the blood–brain barrier can be useful in this context and promising for the treatment of brain diseases.^150,151^ For example, a Trojan horse trick has been used to counteract drug resistance wherein the drug is hidden inside a DNA capsule and enters the cell like a Trojan horse and prevents the drug from being drained by the cell.^152^ Further, drug-carriers can also bind specifically to receptors on the endothelial cells and enter the brain parenchyma by receptor-mediated transport.^153^ Two important and effective advantages of nanotechnology-assisted delivery to the target organ are the enhanced efficacy of the drug and the reduction of side effects to other organs. Today, different types of metal, lipid, and polymeric nanoparticles have been used in drug delivery to the brain.^154,155^ In neurodegenerative disease, the alteration of the blood–brain barrier and the size of nanoparticles are important factors affecting the release of nanoparticles into the brain parenchyma. Evaluation of nanoparticle toxicity on neurons in clinical and *in vivo* environments is one of the most important challenges pertaining to the deployment of nanotechnology.^156,157^

### 4.2 Neurological Diseases and Role of AXT

#### 4.2.1 Oxidative Stress and Its Assorted Roles in Neurodegenerative Diseases

Oxidative stress is an imbalance between free radicals and the antioxidants in the body resulting in the generation of ROS. Oxidative stress plays an important role in the development and progression of many degenerative diseases such as autoimmune diseases, cancer, heart disease, and diabetes. Notably, AXT plays a very specific role in neurodegenerative inflammatory diseases such as Alzheimer’s, Parkinson’s, Huntington’s, amyotrophic lateral sclerosis, multiple sclerosis, and other processes related to pathological aging.^159,160^ With the increase in life expectancy, the prevalence of neurodegenerative diseases is also increasing, which have various symptoms such as altered mitochondrial function, abnormal accumulation of proteins and proteasomes, and reformed iron metabolism affecting different parts of the brain which can lead to a defective cycle and the onset of cell death.^161^ Factors that produce ROS can damage mitochondria, increase Ca^2+^ levels, inhibit proteasome function, and ultimately lead to neuronal destruction. For physiological reasons, the CNS is believed to be highly sensitive to oxidative stress. The human brain makes up only a small percentage of the total body weight; however, the brain consumes 20% of its basic oxygen consumption. The major ROS involved in the destruction of neurons are superoxide, hydrogen peroxide, and highly active hydroxyl radicals.^162^ Nitric oxide as a high-diffusion biological messenger plays an important role in the physiology of the central nervous system. After production, nitric oxide reacts rapidly with superoxide to produce strong peroxynitrite (ONOO^–^) and hydroxyl radicals; ROS and reactive nitrogen species collectively cause oxidative stress in the nervous system. The CNS is a reservoir of unsaturated lipids that are highly vulnerable to peroxidation and oxidative changes. The double bonds in unsaturated fatty acids are critical sites for attack by free radicals that trigger a chain reaction, thus inflicting damage to their adjacent unsaturated fatty acids.^163^ The brain’s antioxidant defense system is not adequate enough; brain tissue has relatively lower antioxidant activity than other tissues; for example, the brain has 10% of liver’s antioxidant activity.^164^

#### 4.2.2 Inflammation and Brain Diseases

Inflammation in the brain is known as nerve inflammation and can be caused by messages from destroyed neurons in the nervous system, invading germs such as viruses and bacteria, harmful chemicals, and also the deformed proteins (such as beta-amyloid peptides) in the brain.^165^ Two major mechanisms that cause inflammation in the brain are

(1) peripheral inflammation that occurs in the body and can stimulate the brain’s immune system to cause inflammation in the brain tissue and
(2) direct cellular damage to the brain that can trigger inflammation processes.^166^ Neuritis is seen in many pathological conditions such as stroke, infection, and neurodegenerative disorders.^167^ This process is characterized by activation of microglia, increased permeability of the blood–brain barrier, and peripheral immune cell permeability to brain tissue, sequestration of inflammatory cytokines, and ultimately the failure to control inflammation with neuronal injury and death. These processes are not only affected by microglia but also by astrocytes, neurons, and endothelial cells of the brain blood vessels, T cells, and peripheral aliens.^168^ Microglia is part of the immune system and acts like macrophages in other tissues, accounting for ∼10–15% of the brain’s cell population.^168^ In neurodegenerative diseases, microglial cells resemble the M1 phenotype of peripheral macrophages and produce harmful environments for neurons by producing inflammatory cytokines (TNF-α, IL1β, IL-6, NO) and ROS.^169^

#### 4.2.3 Effect of Inflammation-Promoting Factors on Cerebrovascular Endothelial Cells

Peripheral inflammation can affect the brain in several distinct ways. Bacterial lipopolysaccharides are a classic example of the pathogen-associated molecular patterns of pathogen recognition and inflammatory signaling that stimulate the innate immune system.^170,171^ Lipopolysaccharides target cells, express CD14 and TLR4, and by activating intracellular cascades, eventually lead to activation of transcription factors including NFKβ and AP1.^172^ These factors are transmitted to the cell nucleus and transcriptionally trigger inflammatory factors. The activities of iNOS, COX2, and NADPH oxidase are increased, resulting in enhanced production of NO, PGE2, ROS, inflammatory chemokines, and pro-inflammatory cytokines in cerebral vascular endothelial cells.^173,174^ This activates microglia and stimulates astrocytes and initiates inflammatory cascades in the brain tissue. By increasing the expression of adhesion molecules and damage to the blood–brain barrier during inflammation, peripheral macrophages can also enter the brain tissue and promote inflammation in the brain.^175,176^ Systematic injection of lipopolysaccharides also enhances the production and release of aldosterone which overactivates mineralocorticoid receptors in cerebrovascular endothelial cells, thus intensifying the production and release of proinflammatory cytokines.^177,178^ Inflammatory mechanisms that are triggered by damage to brain tissue cells vary, sometimes due to genetic defects and in most cases due to unknown factors, wherein neurodegenerative or autoimmune diseases can play a role. For example, the amyloid-beta peptide, which accumulates in the brain in Alzheimer’s disease, can stimulate inflammatory processes in brain tissue. Other causes of neuritis include stroke, head injury, and direct infection of the brain tissue.^178^ The notion that there is a link between systemic inflammation and dementia first emerged when an increase in inflammatory processes had been observed in post-mortem Alzheimer’s patients. Studies have shown a link between dementia and elevated cytokine levels such as IL-1β, acute phase reactive protein, TNFα, and IL-6.^179^ Furthermore, laboratory studies have shown that the serum and cerebrospinal fluid of Parkinson’s patients have higher levels of IL-1β, TNF-α, and IL-12 as well as CD4^+^ and CD8^+^ lymphocytes, which indicate the activation of peripheral lymphocytes.^180^ The activity of microglia produces large amounts of free radicals, including superoxide, hydrogen peroxide, hydroxyl radicals, and cytokines with cytotoxicity, which damage neurons.^181,182^

#### 4.2.4 AXT and Brain Protection

The pathways of inflammation, oxidative stress, and apoptosis cause the destruction and death of neuronal cells and eventually result in neurodegenerative disorders.^143^ Several direct and indirect mechanisms have been proposed regarding the positive effects of antioxidants on cognitive function improvement as they can affect cognitive function through reduced inflammation, NF-κB regulation, and reduced cytokine production. AXT is a powerful antioxidant with restorative, antiseptic, antiaging, and anti-inflammatory properties and is being used in the treatment of many neurological diseases such as neuropathic pain, Alzheimer’s disease, Parkinson’s disease, autism, depression, etc.^183^ Its unique chemical structure allows it to easily cross the blood–brain barrier and reach the brain, which is the most important target organ for AXT. The ability of AXT to regulate the immune system, reduce inflammation, and treat neurodegenerative diseases has been confirmed.^184^ There have been reports of increased production of IL-6 in the progression of multiple sclerosis disease,^185^ which causes demyelination and neuroinflammation due to its destructive effect on the blood–brain barrier. In a study, it has been found that AXT crosses the blood–brain barrier easily, allowing the carotenoid to protect the CNS against chronic and acute neuronal damage.^98^

Th1 cytokines are involved in the development of MS, and AXT modulates the response of the immune system by shifting the Th1 to Th2 cell response.^186^ According to the obtained data, it has been concluded that AXT, as an oral supplement, has an effective role in the prevention, healing, and reduction of inflammation and neuronal damage caused by multiple sclerosis. The potential of AXT to reduce ischemic damage in the mammalian brain through preventing apoptosis and suppressing ROS has been reported;^187^ it protects against injuries caused by high blood pressure, vascular oxidation, and cerebral thrombosis. Moreover, AXT prevents nerve damage and reduces the risk of stroke by suppressing the ROS and activating the Nrf2-ARE route. Therefore, it may be useful for ischemic susceptible patients to have a protective effect against neurological disorders caused by the toxicity of free radicals.^188^ Accumulation of amyloid-β peptide oligomers decreases the expression of type-2 ryanodine receptors and enhances the production of mitochondrial ROS, which ultimately lead to neuronal cell death and Alzheimer’s disease. AXT is capable of protecting nerve cells against the harmful effects of amyloid-β peptide oligomers by regulating type-2 ryanodine receptor gene expression and thus can be useful in treating Alzheimer’s disease.^103^ This red carotenoid significantly reduces the levels of amyloid-β peptide oligomers, TNF-α, nitrite, and AChE, the oxidative stress, and the activities of GSK-3β and IRS-S307 in the hippocampus and prevents the insulin resistance of the hippocampus involved in Alzheimer’s disease.^189^ A study has shed light on the capability of AXT as a protective agent against progressive Alzheimer’s disease. Pure AXT and its combination with docosahexaenoic acid have been administered to APP/PSEN1 double transgenic mice up to 2 months. The results revealed that the combination had a stronger effect on the regulation of oxidative stress, inflammasome expression and activation, plus reduction of Tau hyper-phosphorylation, and suppression of neuroinflammation in mice than the pure AXT by itself.^190^

High glycosylated hemoglobin levels, acute phase reactive protein, IL-6, and TNF-α increase cognitive impairment in depressed diabetic patients.^191^ On the other hand, several clinical studies suggest that mood disorders can be a risk factor for Alzheimer’s disease.^192^ Recent studies propose that preventing inflammatory reactions in the brain and reducing nerve damage can reduce depression in diabetic mice.^193^ Therefore, reducing inflammatory cytokines appears to be effective in the pathophysiology and treatment of the depressive disorder.^194^ In many studies, natural ingredients have been studied as supplements to improve mood and reduce anxiety and stress by inhibiting inflammation.^195^ Animal studies revealed that the severity of depression has been reduced when mice were treated by oral AXT (25 mg/kg) for 10 weeks.^196^ Also, in some studies, daily intake of 0.2 mg of shrimp oil supplement containing AXT for 7 weeks improved the learning, working memory, and depression.^197^ An increase in survival and proliferation of human adipose-derived stem cells has been observed when AXT is used. The use of AXT can increase the transplantation efficiency of human adipose-derived stem cells in the treatment of MS, which is a debilitating disease of the brain and spinal cord (central nervous system).^198^

## 5 Ocular Delivery System for Medicinal Use of AXT

### 5.1 Eye Physiology, Diseases, and Challenges

**Figure 11:**
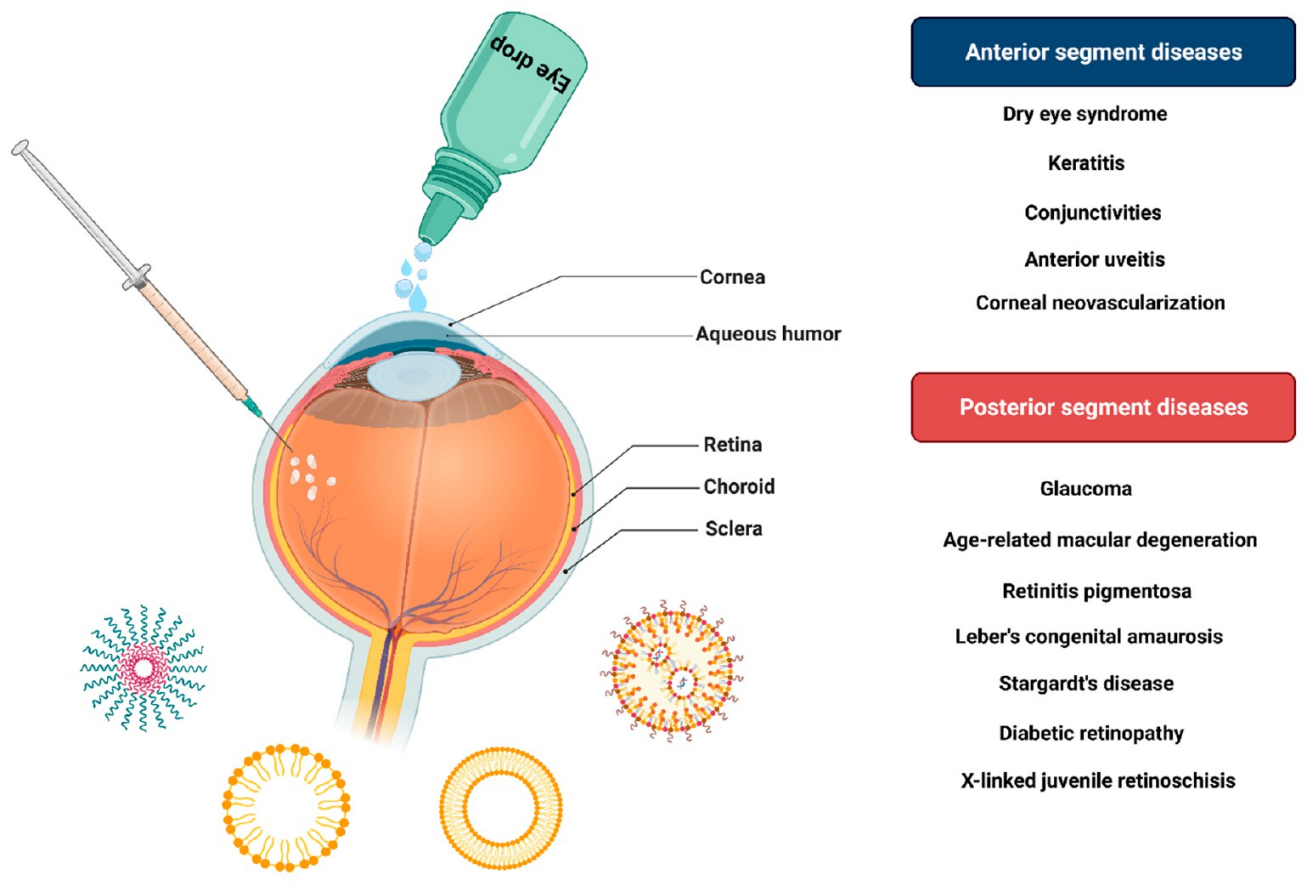
Diseases related to different parts of the eye and various methods of drug delivery to the eye.

Medications used for eye diseases often affect the surface of the eye or its anterior part. The treatment of some diseases such as glaucoma, retinitis pigmentosa, leber congenital amaurosis, stargardt, x-linked juvenile retinoschisis age-related macular degeneration (AMD), and diabetic retinopathy is related to the posterior or back of the eye. Some anatomical structures, including the cornea, sclera, conjunctiva, and retinal epithelium pigment, challengingly limit the effectiveness of the drug delivery to this portion of the eye.^199,200^ Due to protective mechanisms such as tearing and reflex blinking, a small percentage of the prescription drug can be absorbed. Tears wipe away microorganisms and waste materials and even remove drugs from the surface of the eye. Besides, a part of the drug binds to the protein in the tears and thus becomes inactive.^201^ The presence of tight junctions in the corneal epithelium restricts drug delivery to the eye. Because of the 3-layer cornea and also its lipophilic and hydrophilic properties, the drugs that are designed to pass those barriers can reach the target.^202,203^ The eye contact time is about 5 min, which only accounts for about 5% of prescription drugs.^204^ Repeated administration may compensate for the short duration of drug exposure to the cells of the eye, but it may increase the risk of cytotoxicity. Besides, intraocular injection of the short-lived drugs for posterior diseases of the eye is problematic because repeated injections increase the risk of eye-bleeding.^205^ About 40% of the drugs studied for the treatment of eye diseases are low-water-soluble and lipophilic drugs. As a result, it is not possible to use them in the usual formulations with an aqueous base. Therefore, biocompatible and biodegradable nanoparticles are selected for intraocular administration to have an acceptable shelf life and adhesion ability to the mucous membrane (Figure 11**)**.^206−208^ The results of *in vivo* studies have revealed that the nanoparticles have bioadhesive ability which increases the drug’s shelf life and enhances the drug uptake. The use of biodegradable polymers is also a very suitable method for drug delivery to the posterior areas and treatment of chronic eye diseases. By optimizing the surface of nanoparticles, the bioavailability and shelf life of drugs in the eye can be improved.

### 5.2 AXT for Ocular Diseases

**Figure 12:**
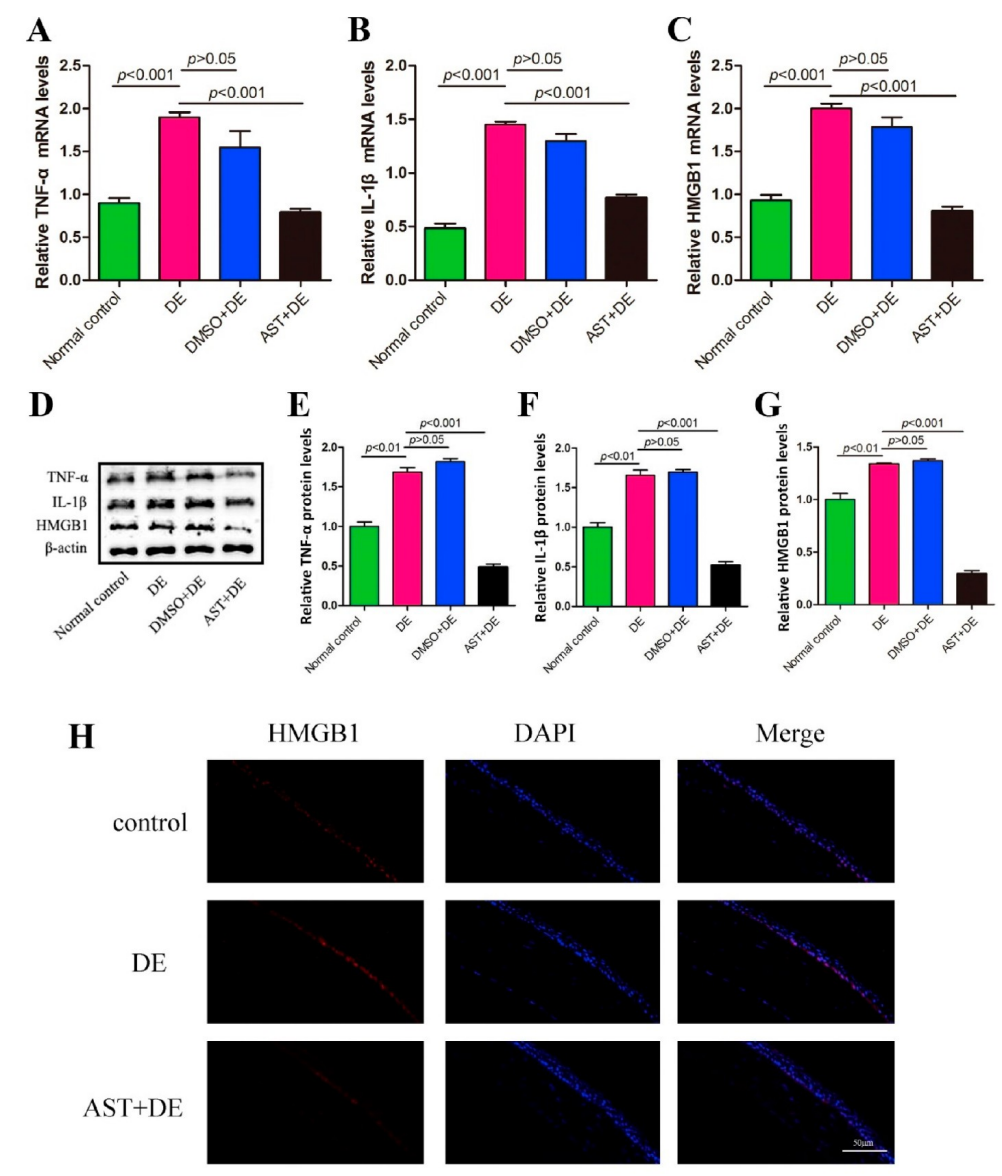
(A**–**C) mRNA expression of TNF-α, IL-1β, and HMGB1. (D**–**G) Protein expression of TNF-α, IL-1β, and HMGB1. (H) Fluorescence images showing the expression of HMGB1 in the corneal epithelium. Reprinted from ref (221) with permission from Elsevier.

AXT helps protect retinal cells against oxidative damage and UV light and relieves symptoms of eye fatigue^108,209^ with validation that AXT inhibits ROS production and retinal cell death.^210^ Retinal ischemia increases NF-κB production and induces retinal inflammation.^211^ In retinal diseases, glia cells play an essential role in inflammation by producing inflammatory cytokines such as IL1β and TNFα.^212^ These cytokines activate transcription of COX2 and iNOS genes, leading to the synthesis of NO and PGE2, which are inflammatory mediators.^213^ AXT inhibits NF-κB activation and expression of COX2 and iNOS.^214^ The topical use of AXT limits the damage caused by the effects of ultraviolet radiation, and also the level of apoptotic cells was significantly lower in the irradiated coronas treated with AXT eye drops; it is reportedly more effective in protecting the ocular surface from UV than the systemic injection.^215^ Nonetheless, AXT reduces inflammation in the retina via reduction in the expression of TNF and IL1β.^186^ This antioxidant reduces apoptosis in retinal ganglion cells as well as retinal pigment epithelium by increasing the expression of p-Akt, p-mTOR, and Nrf2. It also decreases the expression of caspase-3, thus preventing glaucoma and AMD.^210,216^ AXT has a protective effect on the retina and treats injuries caused by the elevated intraocular pressure^217^ and hence inhibits the glaucomatous retinal degeneration.^209^ Nowadays, drug macromolecules as angiogenesis inhibitors including aflibercept, pegaptanib, and ranibizumab with molecular weights of 97, 50, and 48 kDa, respectively, are AMD’s first treatment. These drugs target the endothelial vascular growth factor, which is associated with choroidal neovascularization during AMD.^218^ For efficient delivery of biomolecules to the posterior segment, intrauterine injections are often performed, which have disadvantages such as eye infections, patient discomfort, high intraocular pressure, and retinal artery occlusion.^219^ Since macromolecule drug delivery is still in its infancy, alternative delivery strategies are much sought after. Notably, considerable attention is now focused on ocular delivery of small drugs. AXT as a small molecule can be used to treat eye diseases, especially AMD, which must target the posterior part and cross the barriers.^220^ Besides, AXT could be a potential agent to reduce the ocular inflammation mediators in mice through the mRNA exprssion of TNF-α, IL-1β, and HMGB1 as well as the protein expression of TNF-α, IL-1β, and HMGB1 (Figure 12).^221^

### 5.3 AXT Delivery for Ocular Health

Topical medications such as eye drops, eye ointments, etc. for the treatment of eye diseases have advantages as they are minimally invasive and convenient for patients. However, there are some lingering challenges. Most eye drops are removed within seconds due to obstacles such as limited lacrimal capacity and subsequent tears, particularly in the case of high molecular weight and hydrophilic drugs, which unlike small molecule lipophilic drugs, have very limited permeability. Drug molecules are transported via two pathways (corneal and noncorneal) to reach the anterior and posterior segments, respectively, and both have barriers to drug permeation.^222^ Thus, some issues should be addressed such as the drug’s molecular size and weight, its permeability, hydrophilicity, and hydrophobicity, and above all its delivery system. Topical application of drugs is a preferred route for diseases of the surface or the anterior portion of the eye that affect the cornea or sclera and the lens. For the drug cargo delivery to the posterior ocular segments, there is a need for further investigation to develop appropriate systems or devices to overcome the barriers within the ocular tissue. Among numerous studies, the use of nanotechnology-based drug formulations has been one of the most successful. Developing novel nanoformulations for *in situ* delivery and release of therapeutic molecules can circumvent ocular barriers and reduce systemic side effects. AXT, as a lipid-soluble keto-carotenoid, is used in the treatment of oxidative stress-induced ocular diseases including AMD and dry eye due to aging, allergies, inflammations, etc.^108,183^ Since the retinal epithelial cells are the active site of this drug, delivery to this site is of particular importance. As topical routes, namely eye drops, are more practical and easier to use for patients, it is important to design an appropriate drug delivery system for topical application of AXT, which has poor solubility in aqueous solutions.^223^ Nanosized liposomes are a good choice because they can cover hydrophobic AXT well and alter the drug’s surface charge, followed by delivering the cargo to the desired position in the posterior ocular tissues. AXT-coated liposomes have been applied in an *in vitro* dry eye model, and its effect on reducing cell apoptosis and inhibiting ROS production and aging markers is evident. Moreover, it has been revealed that when positively charged liposomes are applied, AXT delivery to the desired location increased locally. Cationic liposomes exhibit higher affinity toward cells than neutral ones. This higher affinity could make them a suitable candidate as a nanocarrier for drugs such as AXT.^224^ Transportation of drugs via a topical route can be enhanced by mucus-penetrating delivery systems to various ocular tissues beyond the mucus layer; mucus-penetrating nanoparticles have been tested *in vivo* to discern improvement of drug diffusion. The results implied that it enhanced diffusion not only toward the ocular surface but also toward the posterior segments.^225^ Furthermore, some biological molecules such as peptides (as cell-penetrating agent), proteins, monoclonal antibodies, genes, and oligonucleotides can be conjugated to nanoparticles for drug transportation to the posterior segment of the eye.^226^ Nanoparticles and liposomes, nanomicelles, nanosuspensions, and dendrimers are other nanotechnology-based carrier systems which are being studied for ocular delivery therapeutics.^227^ Nevertheless, nanoformulations seem to help in overcoming various ocular barriers better than other delivery systems for AXT. However, there is still room to discover novel drug delivery systems to increase the stability, solubility, and bioavailability of AXT.

## 6 Dermal Delivery of AXT for Skin Protection

### 6.1 Skin Morphology, Barriers, and Penetration Routes

**Figure 13:**
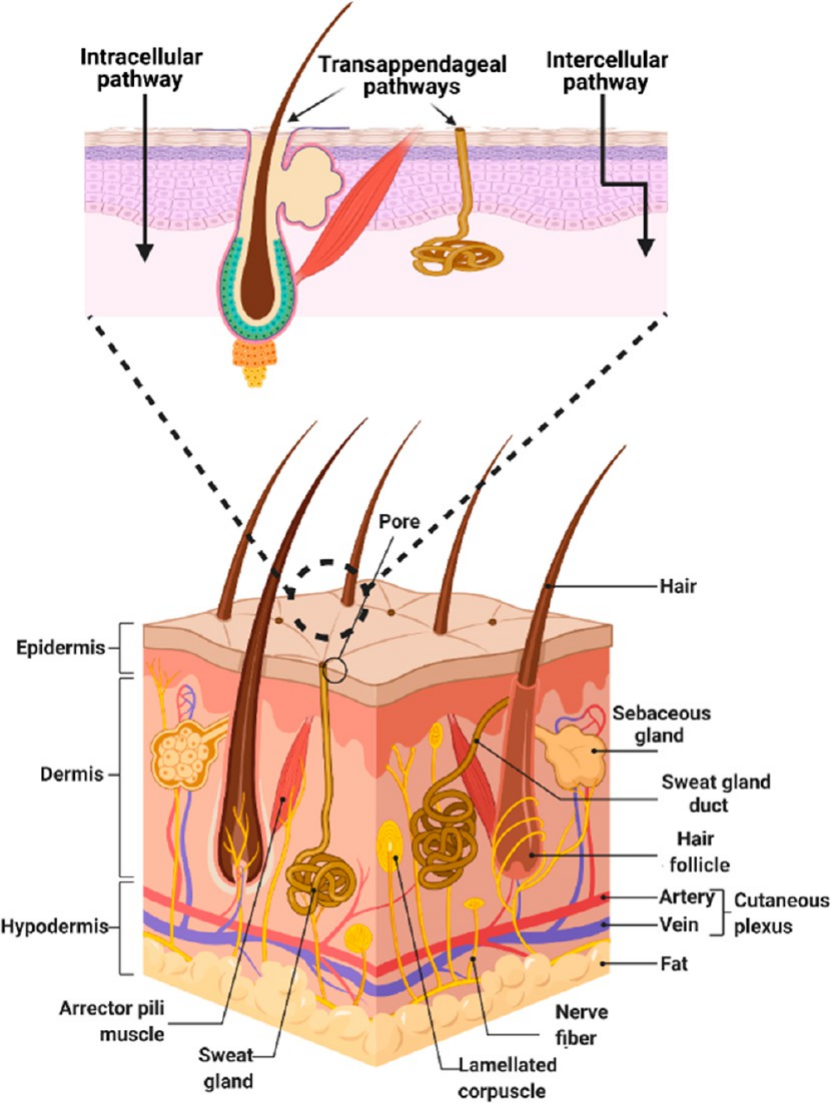
Schematic illustration of skin layers and major skin permeation routes for the delivery of nanoparticles. The first one is the pathway through opening areas of the skin such as sweat glands and hair follicles which leads to a better penetration of the drugs into the skin. Drug molecules diffuse through the phospholipid membranes and cytoplasm of the deceased keratinocytes. In this continuous way bioactive agents pass through the small spaces between the cells of the skin.

Skin is the initial barrier for living creatures against the environment, and the first obstruction to penetrate it is the stratum corneum, the main barricade for drug penetration.^228^ There are two main routes through the skin for the permeation of active substances: trans-appendages and trans-epidermal pathways. The trans-epidermal pathway is responsible for skin permeation and comprises two routes, intercellular (paracellular) and transcellular (polar) pathways (Figure 13).^229^ The intercellular pathway is the major penetration pathway for active antioxidants into the skin and even possibly into deeper areas of the skin.^230^ Nanotechnology is of immense help for successful skin drug delivery. It can control the release of drugs to enhance performance, provide higher drug loading capacity, help attain the physical and chemical stability of the drugs during the time of storage, and prolong the drug delivery, thus improving the drug concentration.^231^ The size of the drug molecule is the first challenge for its penetration due to the 10–40 μm thick stratum corneum wherein drugs with relatively low molecular weight (∼ below 500 g/mol) can reach the dermis.^228^ Besides, the cells present in this layer are haphazardly arranged; therefore, the drug has to travel a long way to cross this layer.^232^ To date, a variety of different physical and chemical approaches for enhancing drug delivery parameters through the skin have been devised; many of them are costly irritants.^233^ The novel nanotechnology-based approach for topical drug delivery with controlled drug release has been recognized as an effective strategy especially for drugs with poor water solubility and short half-life.^234−236^ Besides the role of the nanoparticles and nanocarriers in treatment of skin disorders, they have been widely used in the cosmetic industry; moisturizing creams containing liposomes were first developed ∼40 years ago.^237^ The skin has the most contact with the external environment, and therefore, it demands more care and maintenance. Daily skin care, deploying cosmetics containing nutraceuticals, enhances the skin’s elasticity, texture, and smoothness, thus promoting skin health.^238^ Delivering the drug through the skin by transdermal patches or topical formulations is problematic because of the presence of the stratum corneum; this layer of the epidermis limits the delivery of bioactive molecules with relatively low molecular weight. To overcome these limitations in passing biological barriers, microneedle patches are a promising tool to perforate the stratum corneum.^239^ Microneedles, comprising micro-/miniature-sized needles, are able to deliver cargo into the dermis following a noninvasive route.^228^ However, to date, no study has been undertaken to deliver AXT via microneedles. Hence, there is room for conducting research in microneedle-mediated delivery of AXT.

### 6.2 AXT Delivery for Skin Health

There is a balance between reactive oxygen and nitrogen species generation and antioxidant system activity in living cells. The structure and functionality of normal cells changes when any factor leads to the disruption of this balance.^240^ The disadvantages of excessive oxidative stress for the skin are facial lines, deep wrinkles, dullness and roughness, dry aged skin, and the loss of elasticity.^241^ UV rays can penetrate the skin and create oxidative stress, followed by DNA, protein, and lipid damage, and errors in DNA repair leading to mutation, collagen degradation, wrinkles, erythema, and skin cancer.^242^ AXT enhances skin health through several mechanisms including antioxidant properties, anti-inflammatory effects,^243^ improving immunity,^244^ and the DNA repair effect.^245^ Many studies have evaluated the efficacy of AXT on the skin and demonstrated that it improves skin elasticity, texture, and moisture content and decreases wrinkles and visible signs of aging.^246,247^ Due to the anti-inflammatory and antioxidant properties of AXT, it has been suggested to potentially decrease skin cancer rate;^248^ the cosmetic benefits of AXT have also been investigated by some researchers. In a topical application, a cream containing AXT was used on 11 females’ skin. After 3 weeks, the skin moisture as well as the elasticity of the majority of applicants’ has increased and three females with fine wrinkles showed improvement in their skins. In another study, a group of 49 women of 45–50 years of age were administered 4 mg of AXT for 6 weeks with over 50% of the participants’ skin features, including elasticity and moisture, being improved.^249^ AXT initiates the cellular antioxidant defense system and modulates the Nrf2 pathway, leading to antioxidant response.^250^ The Keap1-Nrf2-ARE signaling pathway is the key antioxidant defense system against oxidative stress. Nrf2 is a key transcription factor that is negatively regulated by Keap1, and its main role is to regulate the cell’s protective responses to oxidative stress. Under basic conditions, most of the Nrf2 molecules in the cytoplasm bind to the Keap1 protein and are destroyed. Oxidative stress reduces Nrf2 degradation by altering specific cysteine codes in Keap1, resulting in the transfer of Nrf2 to the nucleus. It binds to ARE in the promoter region of genes encoding antioxidant enzymes and induces the production of endogenous antioxidant enzymes.^81^ These enzymes include superoxide dismutase, catalase, peroxiredoxin, etc., which play an important role in combating ROS.^251^

**Figure 14:**
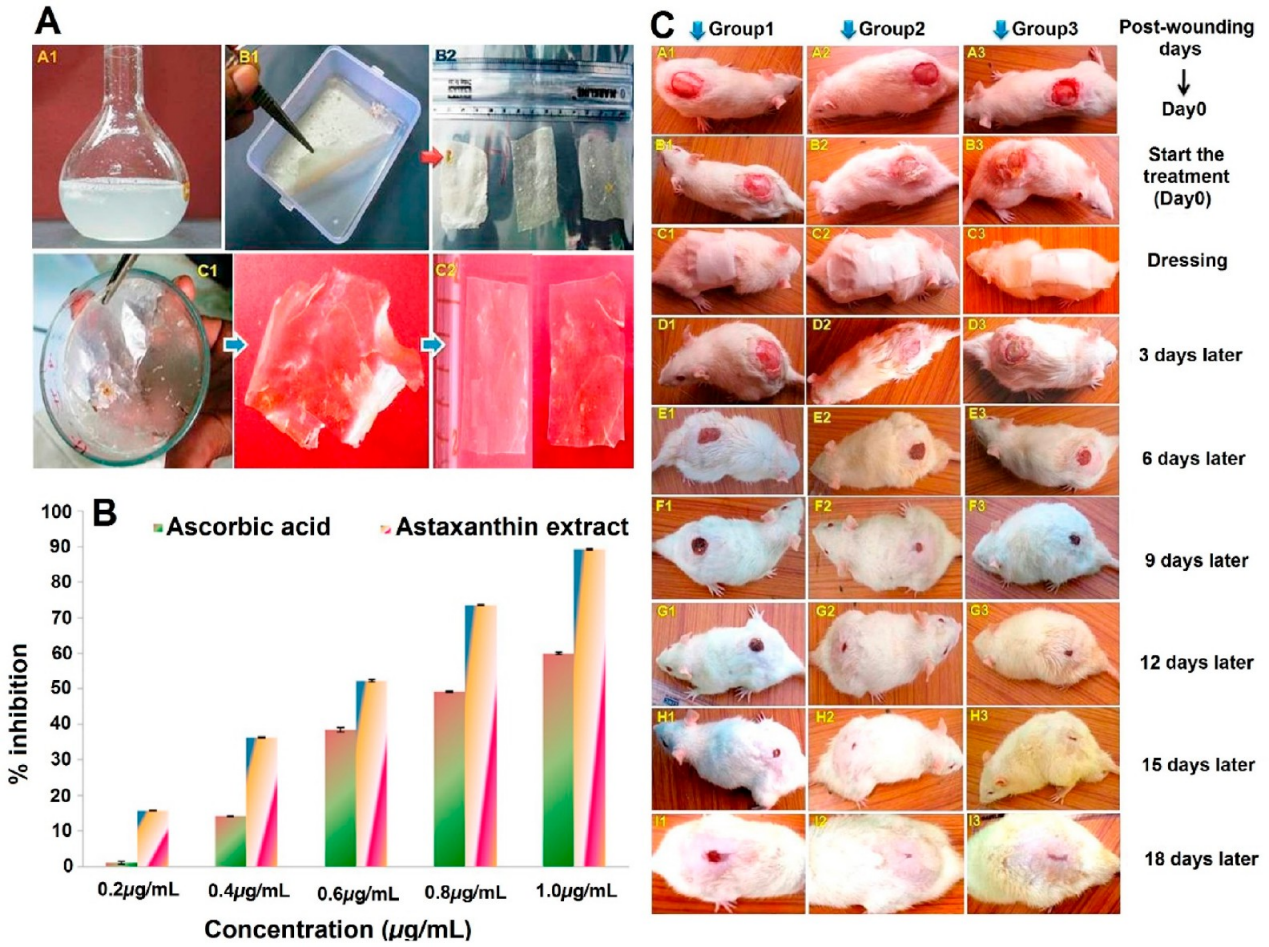
(A) Images attributed to the preparation process of AXT and drug incorporated collagen films extracted from *D. singhalensis* as follows: (A1) Collagen solution, (B1 and C1) films containing AXT and gentamycin in the collagen film solution, (B2 and C2) films before treatment modified square shape (5 × 4 cm) formation. (B) Antioxidation activity (DPPH) of AXT extracted from *D. singhalensis* compared to ascorbic acid in different concentrations. (C) Photographic representation of wound contraction on different postexcision healing days (1–21 days). Group 1 is the control group; group 2 is the AXT incorporated collagen; and group 3 is the gentamicin incorporated collagen. Reprinted from ref (254) with permission from Elsevier.

AXT also affects the function of the immune system; for example, in a study of human lymphocytes, AXT enhanced the immunoglobulin production in response to T cell stimulation. In other studies, it has been proven that AXT enhances immune responses and improves the cytotoxic activity of T and NK cells *in vivo.*(244,252) In a study carried out on healthy female college students, immune system markers including IFN-g and IL-6 production, NK cell cytotoxic activity, and LFA-1 expression have been meaningfully enhanced; cell and humoral immune responses were improved by dietary AXT usage.^253^ It is worth mentioning that the participants with an average age of 21 received daily AXT for a period of 8 weeks and their immune responses were evaluated during a clinical study. In the middle of the experiment, it has been observed that a DNA damage biomarker is decreased by AXT with improvement in young females’ immune response.^253^ Ultraviolet radiation induces the production of reactive oxygen species (ROS) and free radicals such as hydroxyl and singlet oxygen, and these being reactive molecules cause DNA strands breakage and the oxidation of its bases.^245^ Due to AXT’s antioxidant properties, it prevents the accumulation of free radicals, thereby preventing damage to DNA.^104^ In addition, the effect of AXT in the tissue engineering and wound healing in both the *in vitro* and *in vivo* phases showed very promising results. In this manner, combination of AXT with some types of polysaccharides, such as chitosan and collagen, leads to increasing the ratio of wound healing in a fraction of time compared to other types of studies that used only the polysaccharides and/or other types of routine polymeric nanostructures. Comparing the results of the used AXT incorporated collagen with the control group (saline only) and the drug control group (gentamicin incorporated collagen) showed that the AXT could accelerate the wound healing in the rat by up to 50% compared to the two control groups (Figure 14).^254^ Also, it has been reported that AXT may influence the kinetics of DNA repair.^250^ In a study, the protective capability of AXT against UV-induced DNA alterations has been assessed; synthetic AXT hindered DNA damage in human melanocytes and intestinal CaCo-2 cells.^255^ Alterations in extracellular matrix components such as fibrous proteins including collagen, elastin, and glycosaminoglycans lead to skin dryness, wrinkle formation, and the loss of skin elasticity.^256^ UV-induced ROS production stimulating synthesis of matrix metalloproteinases results in extracellular matrix destruction and the loss of collagen. AXT with its antioxidant ability prevents the growth and accumulation of free radicals, and it has been observed to prevent matrix metalloproteinase expression in different cells.^257^ The effects of AXT on the promotion of matrix-metalloproteinase-1 and skin fibroblast elastase on UV-treated human dermal fibroblasts of cultured human dermal fibroblasts have been assessed where AXT decreased the effects of UV radiation on skin.^99^ Pro-inflammatory mediators are reportedly increased during UV radiation, and AXT inhibited the production of inflammatory mediators by blocking NF-κB activation. The effect of AXT on expression of NF-κB p65, IL-6, TNF-α, and IFN-γ has been investigated elsewhere. A total of 32 buffaloes have been supplemented with AXT during a period of 30 days. The inflammatory mediator expression from peripheral blood mononuclear cells is compared to control groups. It turned out that the mRNA expression of IL-6, TNF-α, and IFN-γ decreased in comparison with control groups.^258^ As has been noted, AXT reduces the level of inducible nitric oxide and cyclooxygenase. This property has an important effect on the development of anti-inflammatory drugs.^259^

## 7 AXT and Treatment of Diabetes

**Figure 15:**
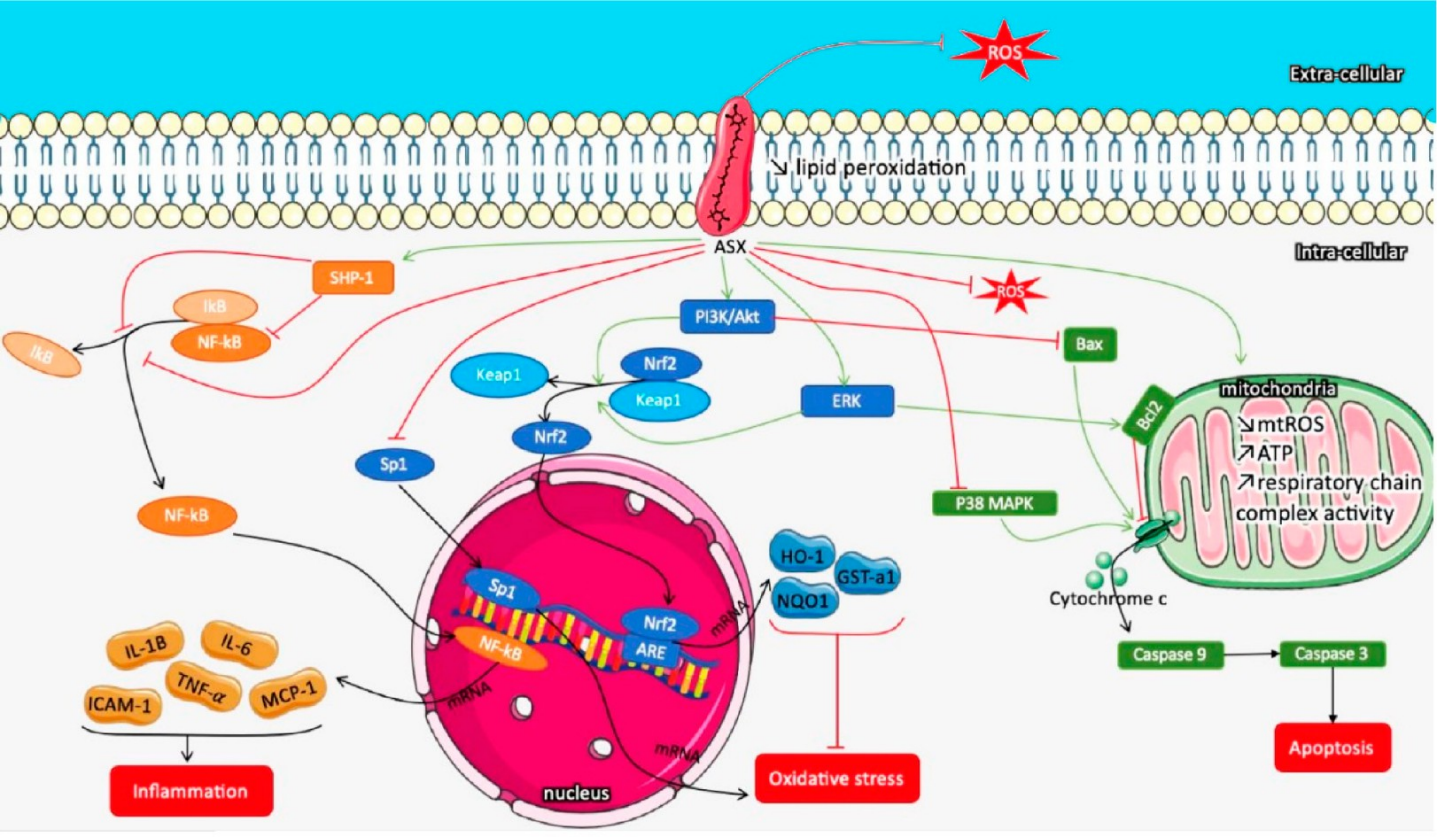
Schematic representation of the molecular pathways implied in the protective potential of astaxanthin (AXT): Due to its membrane penetrance, AXT has both intra- and extracellular ROS scavenging actions. Moreover, in the phospholipid membrane, the AXT polyene chain participates in the reduction of lipid peroxidation. Through the regulation of various pathways, AXT reduces inflammation, oxidative stress, and apoptosis. Red arrows indicate inhibitory action, and green arrows show enhancement action. Reprinted from ref (260) under open access license.

Diabetes mellitus, known as just diabetes among people, refers to a group of metabolic disorders and is recognized with a high blood sugar level over a long time. The number of people dealing with diabetes is about 463 million, and it is expected that this number will increase to 578 million in the next 10 years.^260^ Oxidative stress caused mainly by hyperglycemia-induced ROS is known to have a detrimental effect on the progression of diabetes. AXT with superior antioxidation activity can compensate oxidative damage through various mechanisms—scavenging of free radicals, hampering the peroxidation of lipids, and quenching singlet oxygen. In contrast to other family members of carotenoids, the polar structure of AXT helps the drug molecule to incorporate itself into the cell membrane without disorganizing it, thus leading to a decrease in the hydroperoxide levels of the lipid layer.^261^ Moreover, it has been revealed that AXT is capable of enhancing the mitochondrial activity through reduction of the ROS produced in the mitochondry leading to an increase in the ATP and repiratory activities.^262^Figure 15 indicates the possible mechanisms through which AXT inhibits oxidative-related damages.

Caused by lesions in the renal tubule and glomeruli, diabetic nephropathy is a microvascular complication of diabetes mellitus (type I and II), and the main symptoms recognized are reduction of the glomerular filtration rate, damage in the epithelial cells of the renal tubules, etc.^263^ Oxidative stress is a key factor causing diabetic nephropathy, and AXT with its superior antioxidant property is of particular interest for application in this case. Depending on the stage of diabetes, AXT is effective in treating and reducing its complications. The antidiabetic effects of Astaxanthin have been observed by

- decrease in serum glucose and fructosamine levels in patients using AXT (8 mg daily for 8 weeks)^264^
- melioration of glucose metabolism and lower blood pressure^264^
- lower fasting blood sugar in mice^265^
- decreased MDA (malondialdehyde) in serum and increased SOD activity with glucose reducing effects^266^
- protection of the pancreatic beta-cells against glucose toxicity by increasing their insulin secretion^265^
- prevention of the ER-stress mediation of beta-cell apoptosis^267^
- increased insulin sensitivity and glucose uptake and decreased insulin resistance in high-fat fructose diet (HFFD)-fed mice using AXT for 45 days (6 mg/kg/day) impressed on the insulin signaling pathway^268^
- increased glucose metabolism and tolerance in muscle and decreased insulin resistance in this tissue as well as augmented mitochondrial biogenesis in muscle cells of (high-fat diet) HFD-treated mice^269^
- improved glucose metabolism by affecting AXT on the liver’s metabolic enzymes and increasing the storage of glycogen in the liver^270^
- reduced inflammatory phenomenon and liver dysfunction due to diabetes in (Streptozotocin) STZ-induced diabetic rats by reducing the levels of ROS and AGEs (advanced glycation end products) and reduction of lipid peroxidation in the liver during 18 days of consumption of 50 mg/kg AXT per day^271^

Some complications of diabetes include the following:

(1) **Retinopathy**. A slow-progressive complication of diabetes with increased inflammation, decreased antioxidant enzyme’s functions, numerous metabolic changes in the retina cells, microvascular damage, oxidative stress in the retina and its capillary cells, and activation of the autophagy pathway in retina cells.^272−274^ In a study, the preventive role of AXT on retinopathy in rats has been examined and a decrease in oxidative stress and inflammatory mediators and an increase in antioxidant enzymes were observed.^275^ An *in vitro* experiment on human retinal pigment epithelial cells showed that AXT can reduce the effects of high glucose on cells by decreasing AGEs, ROS, and lipid peroxidation.^276^ Khedher et al. showed the inhibitory effect of AXT on aldolase reductase activity, which is a key enzyme in the pathogenesis of retinopathy.^277^
(2) **Neuropathy**. Adverse effects of this complication are neuronal abnormalities, brain cell apoptosis, hippocampal-based cognitive dysfunction, and neuronal behaviors.^278^ All these problems are due to the activities of oxidative stress, the presence of inflammatory mediators, and the activation of apoptosis-related molecules. Studies show the the protective and melioration effects of AXT administration on neuropathy include increased antioxidant enzymes’ activity, reduced level of inflammatory molecules, protection of cells from apoptosis,^279^ improved neuronal behaviors in STZ mice,^98^ and attenuated cognitive deficit by inhibition of oxidative stress and inflammation in diabetic mice.^280^
(3) **Cardiovascular Effects**. They are diabetes-related disorders caused by thrombosis, arteriosclerosis, vascular damage, and platelet aggregation that all are the result of high glucose and oxidative stress.^281,282^ AXT reduces these effects by reduction of oxidative stress and inflammation as it showed anti-inflammatory and anticoagulatory effects,^283^ regulation of redox reactions, control and regulation of vasoconstriction, blood pressure, and blood fluidity,^284^ and reduction of the LDL level.^285^
(4) **Nephropathy**. Nephroprotective effects of AXT are observed by increased urinary albumin and decreased oxidative stress markers in db/db mice with 12 weeks of AXT administration,^286^ inhibition of COX-2, MCP-1, TGFB, and ROS production in glomerular mesangial high-glucose-stimulated cells,^287^ normalization of creatinine and uric acid levels, reduction of urea and glomerular hypertrophy in diabetic rats and improvement of renal dysfunction,^288^ increase in the expression of antioxidant enzymes, and maintaining the antioxidant status of the kidneys and plasma, which reduce the renal complications of diabetes^289^ and prevent renal fibrosis by reducing the accumulation of ECM components and protection against oxidative damage by activation of transcription factor Nrf 2-ARE.^290^

**Figure 16:**
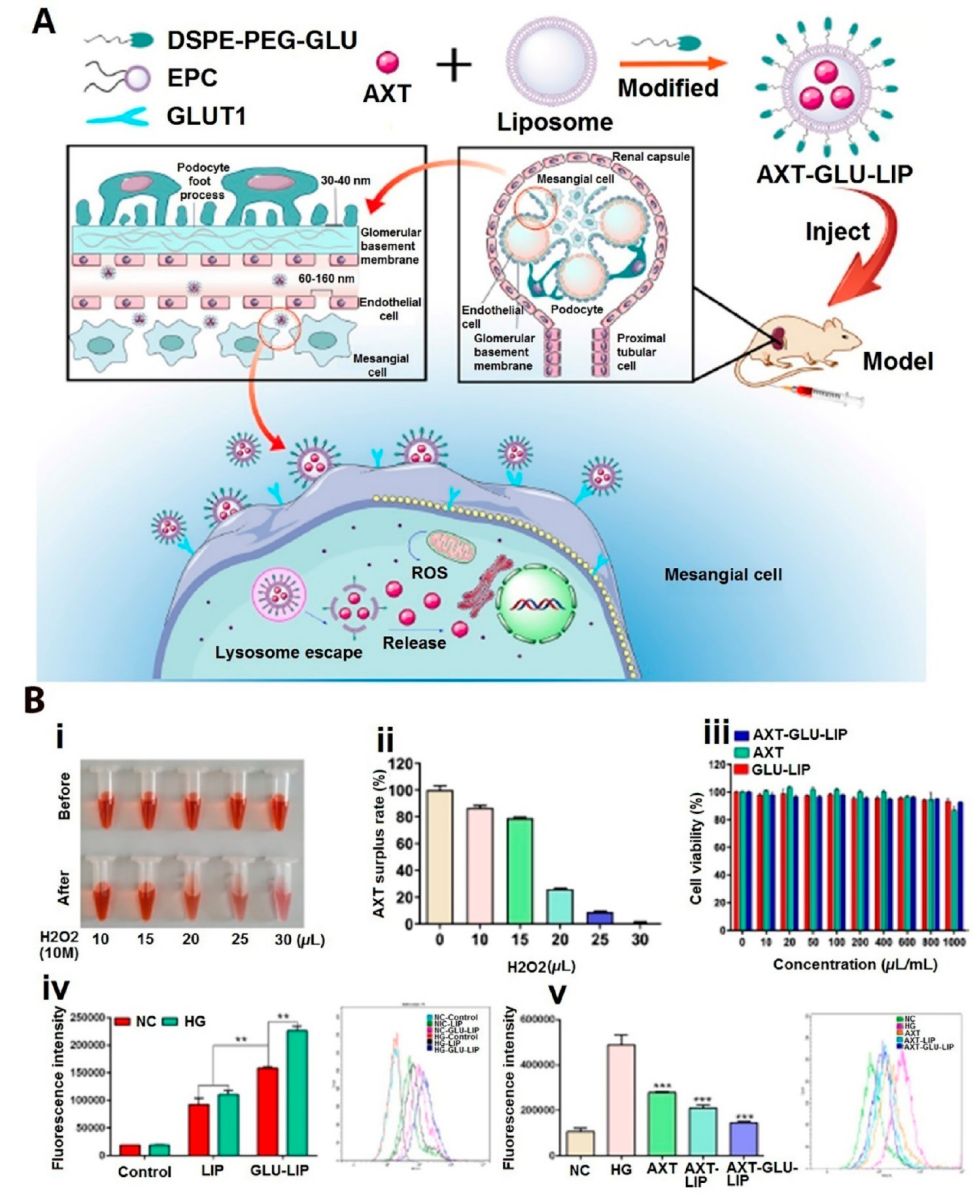
(A) Schematic on the targeting of glomerular mesangial cells through the glucose ligand-modified liposome encapsulating AXT. (B) AXT antioxidative activities (i), the surplus rate of AXT in H_2_O_2_ scavenging (ii), the cell viability of GLU-LIP, AXT, and AXT-GLU-LIP samples in the exposure of human renal mesangial cells (HRMCs) at different concentrations for 24 h (iii), the cellular uptake of DiO-labeled samples through HRMCs (iv), the level of ROS for different samples in the exposure of HRMCs (v). The *p* values, including *p < 0.05, **p < 0.01, and ***p < 0.001, represent a significant difference between the samples and HG. Abbreviations: 1,2-distearoyl-*sn*-glycero-3-phosphatidylethanolamine (DSPE), liposome (LIP), glocuse ligand (GLU), yolk lecithin (EPC), diabetic cell model (HG), negative control (NC), and glucose transporter 1 (GLUT1). Reprinted from ref (292) with permission from Elsevier.

However, the drug’s poor solubility and stability negatively affect its antioxidation capability and bioavailability. A recent study targeted diabetic nephropathy through a drug delivery system comprising liposome encapsulating AXT with the aim of designing a smart delivery system targeting glomerular mesangial cells based on glucose transporter 1 which reportedly plays a significant role in transporting glucose to glomerular mesangial cells.^291^ The glucose-modified liposome encapsulating AXT can successfully penetrate through glucose transporter 1 of the glomerular mesangial cell membrane, and the drug delivery system efficiently scavenged ROS generated by oxidative stress.^292^ Moreover, the AXT release study has been accomplished at different pH’s; the acidic medium exempilified the lysosome environment, while the phosphate buffer saline +10% fetal bovine serum represented the blood environment. The liposomes exhibited a faster release in the acidic environment and a better protection of drug molecules. Figure 16 indicates the physicochemical and biological properties of liposome encapsulating AXT *in vitro* plus a schematic showing how the glucose ligand drug delivery can reach the mesangial cells.

## 8 Therapeutic AXT Delivery for Other Disorders—Cancer

**Figure 17:**
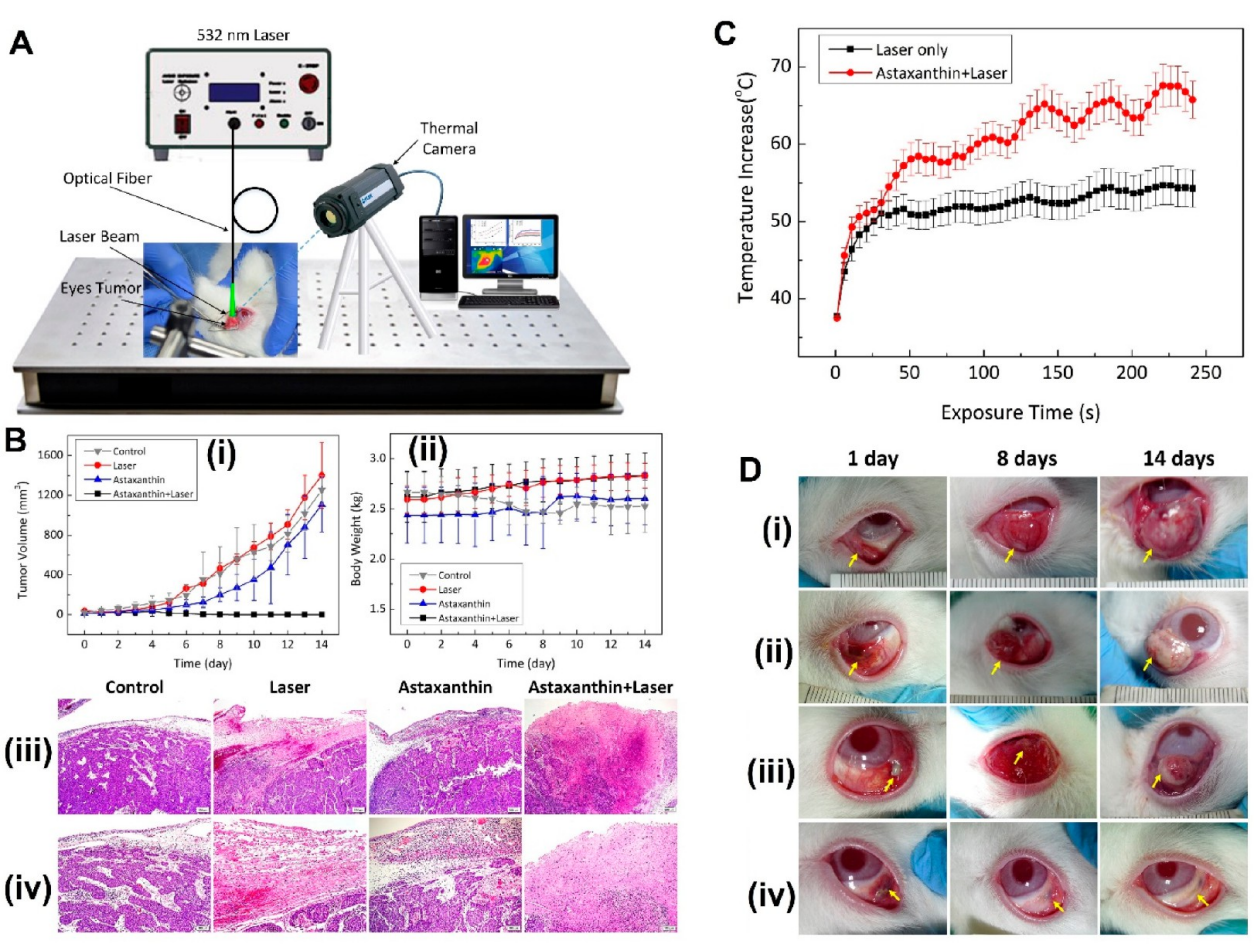
(A) Experimental setup for AXT-induced photothermal therapy; (B) the tumor volume (i), rabbits’ body weight (ii), and H&E staining images at ×40 (iii) and ×100 (iv) of the samples; (C) the *in vitro* assessment of temperature change when NIR with a wavelength of 532 nm had been irradiated; (D) treatment of eye tumors up to 14 days: control (i), tumors treated with the laser alone for 4 min at 532 nm and 0.11 W cm^–2^ (ii), injected AXT solution (300 μg mL^–1^) without being exposed to laser irradiation (iii), tumors treated with both AXT injection followed by laser irradiation (532 nm and 0.11 W cm^–2^ for 4 min) (iv). Reprinted from^306^ with permission from Public Library of Science.

Cancer essentially means the growth of a malignant cell. Human malignancies are the result of a set of distinct genetic events. These changes occur in genes that affect cell cycle control, cell survival, cell movement, and angiogenesis. The entry and progression of a cell through the cell cycle are accompanied by changes in the amount and activity of a family of proteins called cyclins. The amount of different cyclins increases at certain stages of the cell cycle, and due to this enhancement, activation of E/CDK2, D/CDK6, and D/CDK4 cyclins takes place, which causes RB phosphorylation, resulting in cell proliferation. Cell proliferation occurs spontaneously when cell cycle-directing genes are impaired due to mutation or amplification. For instance, activation of cyclin D1, which transpires due to mutation, accelerates cell proliferation by facilitating RB phosphorylation.^293^ Studies show that AXT stops the cell cycle at the stage of G0/G1 and prevents the expression of cyclin D1, by increasing the expression of p53, p27, and p21WAF-1/CLP1 at the same time. One way cells escape cancer is to choose death, i.e., apoptosis. The degradation of the nuclear membrane and cytoplasm of cells and organelles leads to fragmentation of cells which are then rapidly ingested by phagocytes and abducted from the environment. Several genes play important roles in apoptosis, including Bim, Bcl-2, Bcl-XL, Bak, Bax, Bad, p53, and Mcl-1. The proteins Mcl-1, Bcl-2, and Bcl-XL work together to act against apoptosis, while the proteins Bim, Bad, Bak, and Bax play a function in apoptosis.^294−296^ Studies have shown that AXT reduces the expression of anti-apoptotic and increases the expression of pro-apoptotic proteins, promoting the release of cytochrome c and Smac/Diablo into the cytoplasm. Bcl-2 causes the release of cytochrome c from mitochondria, which leads to the activation of caspase-9 and then caspase-3. AXT induces mitochondrial apoptosis in cells through caspases, leading to cancer cell death.^297,298^ AXT exerts antiproliferative effects by increasing the expression of Bax and caspase 3 and decreasing the expression of malondialdehyde and bcl2 in the LS-180 cell line.^299,300^ AXT can treat prostate cancer by inhibiting alpha-reductase enzyme function.^301^ Many studies have pointed to the anticancer role of AXT in prostate, liver, colon, lung, breast, and other cancers.^302−304^ At present, a large number of drug delivery systems comprise nanoparticles, and various materials have been used as drug stimulants or enhancers to improve the effectiveness of the treatment and the durability and stability as well as the safety of anticancer drugs. AXT as a biological molecule can reduce metal salts to form nanoparticles that are suitable for treatment in biological systems; production of gold nanoparticles (Au NPs) with AXT as a natural reducing agent has been assessed. The cytotoxic effect of prepared nanostructures against human breast cancer cells (MDA-MB-231) has been evaluated through a tetrazolium-based assay; AXT-Au NPs display a strong cytotoxic effect against cancer cells, and apoptotic morphology has been detected in the treated cells. The AXT reduced Au NPs, on the other hand, have the potential to act as a promising agent in the field of photobased diagnosis and therapy as they display an interesting UV–vis absorption peak in the near-infrared region that is essential in photobased diagnosis and therapy. A near infrared region laser can penetrate into tissue effectively, and nanoparticles can convert this light into thermal energy, which is applied in photothermal therapy.^305^ It is interesting to note that AXT alone has a photocatalytic property by which it can turn light into heat without any need for an additional photothermal agent. This property has been exploited to eradicate eye tumors through photothermal therapy. An increase in the local heat of tumors has been observed once the near-infrared is applied. The obtained results clearly showed that the AXT is a very promising candidate for the treatment of any type of cancer through photothermal therapy as depicted in Figure 17.

Early detection of cancer can significantly increase the likelihood of successful treatment. Such imaging tests can have a significant impact on cancer diagnosis. Photoacoustic imaging is a hybrid imaging technique based on the photoacoustic effect with high resolution and sensitivity, and it can be used to diagnose different stages of cancer. Compared to other common methods of tumor imaging, it is more economical and has better contrast in tumor diagnosis.^307^ AXT, with an absorption peak at 490 nm, can be used as a potential photoabsorbing agent to enhance photoacoustic responses in targeting cancerous tumors.^308,309^ Nguyen et al. demonstrated that AXT can be employed as an exogenous photoacoustic biocompatible contrast agent to recognize the size and the location of bladder tumors.^310^ Also, Bharathiraja et al. synthesized polypyrrole nanoparticles using AXT-conjugated bovine serum albumin as an optical contrast agent for photobased therapy and cancer detection. In another study, an AXT-alpha tocopherol nanoemulsion has been synthesized by spontaneous and ultrasonication emulsification methods and its effect examined on three different types of cancer cells; it has significant anticancer potential against different cancer cells and exhibits antimicrobial and wound healing properties.^305^

Additionally, some researchers have examined the use of solid lipid nanoparticles as oral delivery systems for vitamins and their analogs because they are biocompatible with the lipid matrix (comprising triglycerides, fatty acids, or glycerol esters) and are readily degraded *in vivo*; AXT, being a natural carotenoid, works against several disorders and is more potent than β-carotene and vitamin E.^5^ However, its use in oral formulations is limited due to its light sensitivity, decomposition in the presence of oxygen, and poor water solubility. Therefore, AXT has been entrapped into solid lipid nanoparticles to improve its bioavailability.^311^ A drug delivery system based on Tween 20 esters and glycerol has been developed for AXT delivery with the average diameter of these solid lipid nanoparticles being 163–167 nm, while the encapsulation percentage was ∼89%. The results reveal that solid lipid nanoparticles caused the long-term release of AXT in GI simulated juices.^312^ In another study, AXT-loaded colloidal particles have been developed to address the limiting factors of AXT for oral drug delivery applications via chitosan oligosaccharide-coated poly(lactic-*co*-glycolic acid) wherein the drug molecules are loaded. Notably, two types of poly(lactic-*co*-glycolic acid) with different lactide to glycolide ratios have been tested (50:50 and 25:75, respectively), and the physicochemical, drug delivery potential, and biological properties have been assessed *in vitro*. Coating of chitosan oligosaccharides made the drug delivery system pH-responsive, and the release rate is increased when the pH of the medium turned to acidic. In contrast to pure AXT and noncoated AXT-loaded poly(lactic-*co*-glycolic acid) samples, the chitosan oligosaccharide coating led to a good dispersity in water at room temperature and enhanced bioavailability which is highly beneficial for drug delivery applications.^313^Table 3 presents some examples of AXT-loaded nanocarriers for different biomedical applications.

**Table 3:** Different AXT-Loaded Nanocarriers Targted for Various Organs^a^

**organ**	**indication**	**nanocomposite drug delivery**		**activity**	**route**	**ref**
**skin**		AXT-NANE		enhance transformation of stratum corneum and permeation of AXT	dermal delivery	(314)
**skin**	wound in diabetic individuals	AXT-TP-KC NEs		accelerate wound healing/control of hyperglycemia	transdermal administration	(315)
**eye**	inherited retinal degeneration (RD)	AXT- polysorbate 20 NEs		eliminate the abnormities in visual signal transmission and visual impairments	oral administration	(316)
**brain**	OxyHb-induced neuronal damage/subarachnoid hemorrhage	AXT-Fe_3_O_4_ -Tf-PEG NPs		neuroprotective	not mentioned	(131)
**brain**		AXT-Tf- PEG-Fe_3_O_4_ NPs		neuroprotective	not mentioned	(131)
**brain**	neurological disorders	AXT-SLNs		neuroprotective	nasal drug delivery	(317)
**liver**	alcohol-induced hepatic injury	AXT-DC NPs	mice	hepatoprotective	oral administration	(318)
**liver**	acute hepatotoxicity	nanoliposomes	mice	hepatoprotective	oral administration	(79)
**liver**	alcoholic liver fibrosis	nanoliposomes	mice	hepatoprotective	oral administration	(319)

a Abbreviations: AXTDC NPs, astaxanthin-DNA/chitosan nanoparticles; SLNs, solid lipid nanoparticles; AXT- Fe_3_O_4_-Tf- PEG NPs, astaxanthin/Fe_3_O_4_/transferrin/PEG nanoparticles; NLCs and CDs, nanoscaled lipid carriers and cyclodextrins; NE, nanoemulsion; NLC, nanostructured lipid carriers; AXT-TP-KC NEs, astaxanthin/alphatocopherol/κ-carrageenan nanoemulsion

## 9 AXT from Bench to Bedside

In addition to medicine, AXT has many applications in a variety of industrial fields. This major microalgal (*Haematococcus)* carotenoid is used for the cosmetic, food, nutraceutical, and aqua-food industries, among others. Commercially, there is a high demand and very competitive market among the producer companies for the production of this pigment and its derivatives. The AXT market for animal feed and nutraceuticals was $300 million and $30 million, respectively, in the year 2009.^320^ In 2018, this market surpassed USD 600 million,^321^ and it exceeded USD 650 million in 2020 (Global Market Insights: https://www.gminsights.com/industry-analysis/astaxanthin-market). Based on Global Market Insights, the AXT market size is estimated to grow at over 5.5% CAGR (compound annual growth rate) between 2021 and 2027. Synthetic and natural AXT are two sources of this market. Generally, the consumption of synthetic AXT is in poultry, pet food, and aquaculture applications, and almost 95% of the AXT market is produced by chemical synthesis.^322^ Although the consumption of synthetic AXT is dominant, consumer demand for the effective natural *Haematococcus* astaxanthin has been growing, especially in the nutraceutical industry. Natural AXT is anticipated to reach US$ 770 million (with the production of 190 t) by 2024, at growth over CAGR of 7.7%.^320^ The AXT market has displayed steady growth since 2014, and its global market size is predicted to reach 3.4 billion USD by 2027, at a CAGR of 16.2%.^323^ It is easily obtainable in various forms of dried meal, powder, oil, and biomass, thus presenting an increase in global pigment sales volume, and will have the most significant global market evolution by 2026.^324^ There is great interest in carotenoids from natural sources, and AXT’s broad applications in food, pharmaceuticals, nutraceuticals, dietary supplements, feed, and personal care products are anticipated to grow.

## 10 Future Perspective and Remarks

One of the problems for human beings today is dealing with chronic and dangerous diseases. Free radicals and oxidants, in general, are continuously produced in the body of living organisms via various metabolic reactions. In view of the role of free radicals and oxidants in the development and progression of these diseases, their counteractive molecules, antioxidant compounds, are becoming valuable supplements in the human diet. In the last two decades, oxidative stress and antioxidants have become one of the most important and popular research areas among researchers.^325^ Diet supplemented with synthesized chemical antioxidants is considered a treatment for ROS disorders, but research shows that regular use of synthetic antioxidants increases mortality.^326^ Therefore, the hypothesis based on the therapeutic effect of antioxidant conditions *in vitro* does not concur with its effects *in vivo.*(327) Side effects of chemical drugs and their incompatibility with human nature have created special importance for the accurate identification and study of the chemical compounds in medicinal plants, yeasts, algae, and several bacteria including natural antioxidants. Natural antioxidants appear to be a good alternative to synthetic antioxidants as they can effectively fight inflammation and oxidative stress;^328^ developed countries have made the development of healthy foods an important priority. By identifying and using these compounds, while improving diet and reducing diseases, they have contributed to enhanced consumer safety and health as affirmed by clinical studies on the health effects of bioactive compounds.^329^ AXT is a healthy nutrient without toxicity, and due to its strong antioxidant properties, it has been involved in protecting cellular compounds against oxidative damage and in regulating gene expression, inducing cell–cell communication and cell health. On the other hand, its use as a natural antioxidant is limited due to its low bioavailability, sensitivity to environmental conditions, processes, and the gastrointestinal tract, and the lack of a proper drug delivery system. Therefore, considerable research has been undertaken on the use of nanocarriers loaded with AXT for therapeutic applications. Besides, the combination of AXT with other nanomaterials may bring synergistic effect, e.g., antioxidant activity, which can be employed for the treatment of different ailments.^330−334^

## Abbreviations Used

AMD: age-related macular degeneration
AXT: astaxanthin
AuNP: gold nanoparticle
C=O: carbonyl group
CNS: central nervous system
COS: chitosan oligosaccharides
DSPE: distearoyl-sn-glycero-3-phosphatidylethanolamine
DHA: docosahexaenoic acid
GLUT1: glucose transporter 1
HRMCs: human renal mesangial cells
-OH: hydroxyl group
LIP: liposome;
NO: nitric oxide
NOS: nitric oxide synthase
ONOO-: peroxynitrit
ROS: reactive oxygen species
SOD: superoxide dismutase
SLN: solid lipid nanoparticles
